# Disposable electrochemical panel immunosensing systems for the simultaneous detection of potential biomarkers of ovarian cancer

**DOI:** 10.1007/s00604-025-07820-8

**Published:** 2026-01-10

**Authors:** Melike Bilgi Kamaç, Ayşenur Yılmaz Kabaca, Merve Yılmaz Çılçar, Muhammed Altun, Mustafa Kemal Sezgintürk

**Affiliations:** 1 Faculty of Science, Chemistry Department, Çankırı Karatekin University, Çankırı, 18100 Türkiye; 2 Şabanözü Vocational School, Department of Medical Laboratory Techniques, Çankırı Karatekin University, Çankırı, 18000 Türkiye; 3 Eldivan Vocational School of Health Services, Department of First and Emergency, Çankırı Karatekin University, Çankırı, 18000 Türkiye; 4 Faculty of Engineering, Bioengineering Department, Çanakkale Onsekiz Mart University, Çanakkale, 17000 Türkiye

**Keywords:** Panel immunosensor, Ovarian cancer, AGR2, GLY, FOLR1, SMRP

## Abstract

Rapid, low-cost, practical, disposable electrochemical panel immunosensor systems were developed for the individual and simultaneous determination of anterior gradient-2 protein (AGR2), folate receptor alpha (FOLR1), glycodelin (GLY), and soluble mesothelin-associated protein (SMRP), which are significantly increased in physiological fluids, particularly in ovarian cancer, and are potential biomarkers in the diagnosis of specific cancer types. To reduce the cost of the panel immunosensor system, the electrodes were hand-fabricated (HSPE), and then the HSPE surfaces were modified with gold nanoparticles (AuNPs). Surface analyses (SEM, XPS, FTIR) have confirmed that the sensor was manufactured robustly. The electrochemical characterization and analysis of the immunosensors were performed using electrochemical impedance spectroscopy, cyclic voltammetry, and differential pulse voltammetry methods. Optimal operating conditions (antibody concentration, antigen, and antibody incubation times) were determined for the prepared single immunosensors. Detection limits, linear detection ranges, selectivity, shelf lives, repeatability, and reproducibility of single and panel immunosensors were determined. The clinical validity of the multi-platform was confirmed by recoveries of over 95% in human serum samples and by ELISA. In this study, low-cost electrochemical panel systems that enable the simultaneous determination of AGR2, GLY, FOLR1, and SMRP biomarkers with high selectivity and accuracy, a wide linear range (1-500 pg mL⁻¹), low detection limits, and good reproducibility were produced for the first time.

**Supplementary Information:**

The online version contains supplementary material available at 10.1007/s00604-025-07820-8.

## Research Highlights

Low-cost immunosensor enables rapid multiplex cancer biomarker detection.

AuNP-modified HSPEs deliver high sensitivity across wide linear ranges.

Surface analyses (SEM, XPS, FTIR) confirm robust sensor fabrication.

Multiplex platform matches ELISA accuracy with low detection limits.

High recoveries in human serum validate clinical diagnostic potential.

**Graphical Abstract:**

**Supplementary Information:**

The online version contains supplementary material available at 10.1007/s00604-025-07820-8.

## Graphical abstract

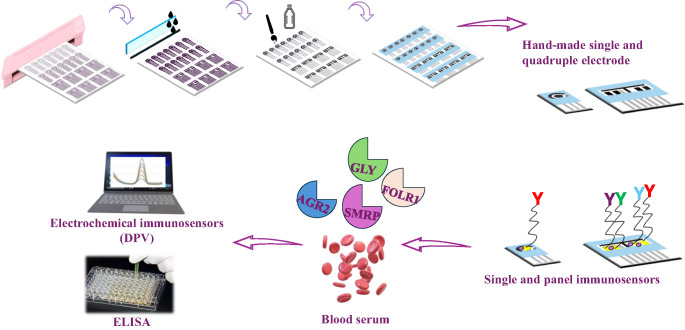

## Introduction

Cancer is one of the most common diseases in the world. According to 2022 GLOBOCAN data, 19,976,499 cancer cases were identified across both genders and all cancer types [1]. It is projected that there will be 2,041,910 new cancer cases and 618,120 cancer deaths in the United States by 2025 [2]. 2022 GLOBOCAN data show that there were 9,664,889 cancer cases and 4,313,548 deaths from female cancers in women [3]. For ovarian cancer, the number of cases was 324,603, and the number of deaths was 206,956 [4]. The earlier cancer is detected, the earlier intervention is possible. It is known that no single cancer biomarker is specific or sensitive enough to diagnose a particular type of cancer, leading to false-positive and false-negative results [5, 6]. For example, due to the diversity of breast cancers, a diagnosis based on only one type of cancer biomarker is often inaccurate [7]. It has been reported that simultaneous detection of breast cancer biomarkers, including carbohydrate antigen 15 − 3, carbohydrate antigen 125, and carcinoembryonic antigen, can greatly improve diagnostic accuracy [6]. Therefore, simultaneous detection of cancer biomarkers is crucial to achieve a reliable diagnosis of cancer [8]. Compared with traditional single-analyte detection, simultaneous multi-analyte detection has been observed to have many advantages, such as fast analysis time, small sample volume, and high accuracy [8–10]. As a result, it is important to produce panel biosensor systems that can detect multiple cancer biomarkers simultaneously [6, 11]– [12].

Anterior gradient-2 protein (AGR2), a member of the protein disulfide isomerase family, has been associated with poor prognosis, particularly with increased expression in breast, ovarian, pancreatic, prostate, and lung cancers, and has been proposed as a biomarker. A clinical study using multiple biomarkers for the early diagnosis of ovarian cancer reported that AGR2 could be used as a biomarker for early ovarian cancer detection [13]– [14]. There is only one study on an electrochemical AGR2 biosensor, and in this study, a label-free AGR2 immunosensing platform prepared with a gold electrode was proposed for the determination of AGR2 [15]. Glycodelin (GLY) is a 47 kDa glycoprotein whose levels increase in diseases such as endometriosis and ovarian cancer, and is a potential marker in stage I-II diagnosis. In a clinical study monitoring GLY level in ovarian cancer patients, higher levels of GLY were detected in the blood serum of patients compared to benign patients [16]– [17]. There is only one study on electrochemical GLY biosensors, and in this study, a label-free GLY immunosensing platform prepared with a gold disk electrode was proposed for the determination of GLY [18]. Folate receptor alpha (FOLR1) is a 40 kDa membrane glycoprotein involved in folate transport. While expressed limitedly in healthy tissues, it is overexpressed in various tumors, particularly ovarian cancer. FOLR1 expression is not altered by chemotherapy [19]. Therefore, it has been proposed as an ideal marker for targeted therapies and targeted imaging in ovarian cancer patients [20]– [21]. Reported FOLR1 biosensors are predominantly DNA-based [22]. These proposed DNA-based FOLR1 biosensors are time-consuming and costly. There are only two studies in the literature on antibody-based electrochemical FOLR1 biosensors [23]– [24], and further studies are needed. Soluble mesothelin-related protein (SMRP) is a glycoprotein that is overexpressed in some types of cancer, especially mesothelioma (lung cancer) [25]. SMRP is expressed by normal mesothelial cells [26]. However, it is highly overexpressed in cancer types such as malignant mesothelioma [27], pancreatic [28, 29], ovarian carcinoma [30, 31], some gastrointestinal [32], or pulmonary carcinomas [33, 34]. SMRP is highly increased in the blood of patients with mesothelioma or ovarian tumors [ 35]. It has been reported that it increases the sensitivity and specificity in the diagnosis of ovarian cancer in combination with CA125 [ 35]. Clinical studies have reported that SMRP can be used as a biomarker in early-stage ovarian cancer patients [36, 37]. We have not identified any studies on electrochemical SMRP biosensors.

In particular, elevated levels of AGR2, GLY, FOLR1, and SMRP in ovarian cancer suggest that simultaneous measurement of these markers may enable early diagnosis. Current diagnostic methods (ELISA, RIA, CLIA, etc.) are time-consuming, costly, and unsuitable for analyzing multiple biomarkers. These limitations necessitate the development of rapid, sensitive, and cost-effective alternatives suitable for point-of-care (POC) testing. Electrochemical immunosensors are a promising approach to address these needs, offering advantages such as high selectivity, low detection limit, and miniaturization.

In this study, electrochemical-based panel immunosensors were prepared for the simultaneous determination of four potential biomarkers of ovarian cancer (AGR2, GLY, FOLR1, and SMRP). For this purpose, carbon-based single and quadruple HSPE were first fabricated and modified with AuNPs under optimum conditions to increase electronic conductivity and ensure stable antibody immobilization. Individual AGR2, FOLR1, GLY, and SMRP immunosensors were prepared using single HSPEs, and optimal operating conditions (antibody concentration, antigen, and antibody incubation times) were determined. The detection limits, linear detection ranges, selectivity, shelf lives, and reproducibility of the single and panel immunosensors were determined, and their accuracies were compared with ELISA assays. Applications of the developed single- and panel-immunosensors in real samples were performed by analyzing target antigens in commercial human serum samples.

## Experimental section

All employed materials, reagents, instruments, and electrochemical measurement parameters are accessible in the Supplementary File.

### Production of handmade electrodes

**Fig. 1:**
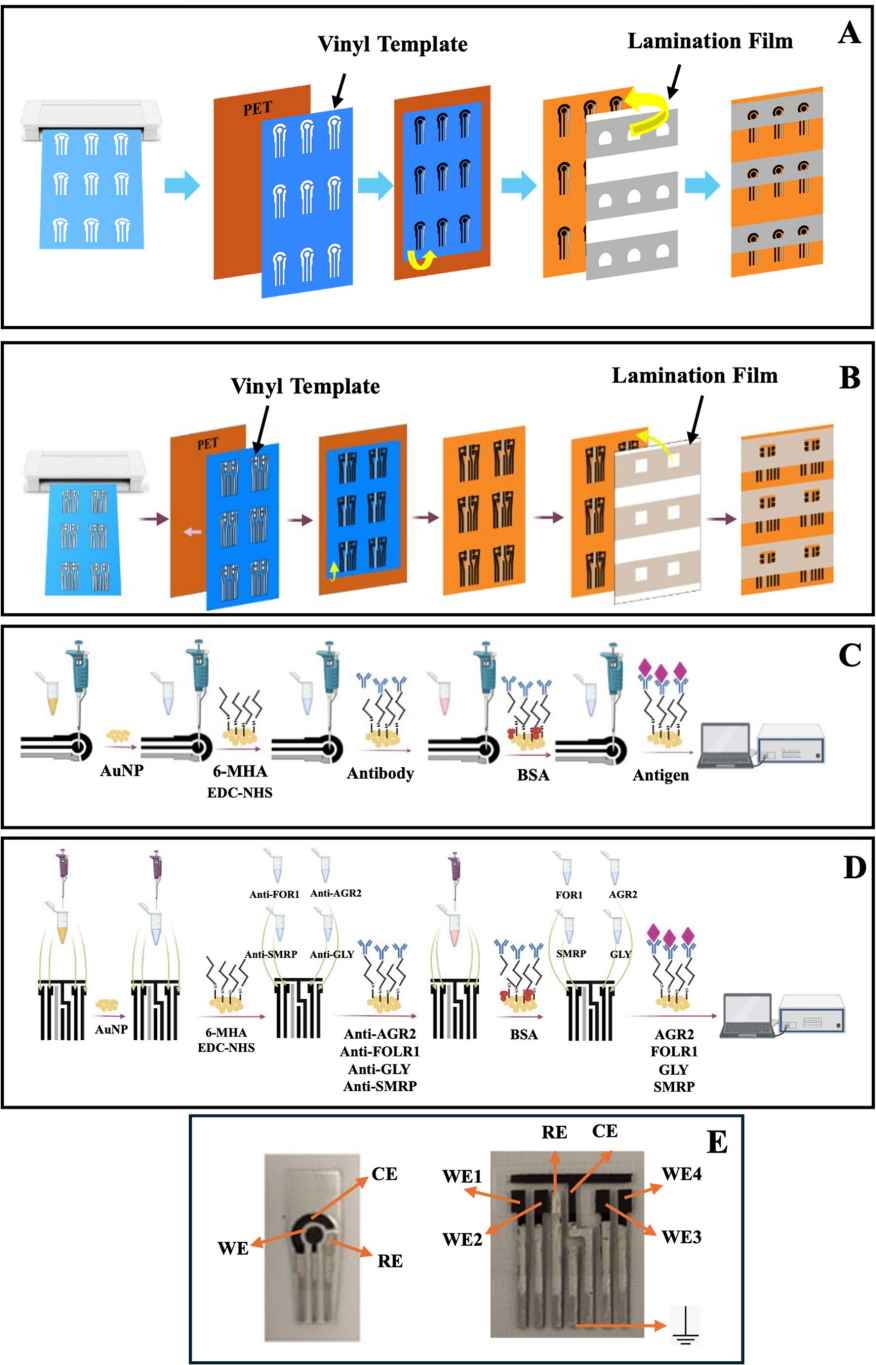
Schematic demonstration of the preparation steps of single **(A)** and quadruple **(B)** HSPEs and for single **(C)** and panel **(D)** immunosensors; photographic images of single and quadrupole HSPEs **(E)**

Using commercial DropSens single (DRP-110) and quadruple (DRP-4W110-U20) electrodes (SPEs) as references, HSPEs were produced by modifying the production method reported in the literature [ 38]. Chemical, morphological, and electrochemical characterizations of the produced HSPEs were performed, and the results were compared with data obtained from SPEs. Schematic demonstration of the preparation steps of single and quadruple HSPEs is given in Fig. 1A-B. Detailed production steps of the HSPEs are provided in the Supplementary Files (Sect. 1.4).

### Preparation of the immunosensors

To prepare single and panel immunosensors, the surface of HSPEs was first modified with AuNP. To form AuNP on HSPEs, HAuCl_4_ solution (100 µL of 4 mM in pH 7.0 PBS) was dropped onto the working electrode (WE), and 10 cycles of cyclic voltammetry (CV) (−1.3 V to −0.2 V, 50 mV s^− 1^) were performed [39–43]. Then, to form SAMs, 3 µL of 100 mM 6-mercaptohexanol (6-MHA) solution was dropped and left for 18 h. Then, 5 µL (single) and 3 µL (quadruple) of 0.6 mM EDC/0.1 mM NHS solution were dropped onto the surface of the HSPEs and left for 1 h. 5 µL (single) and 3 µL (quadruple) of monoclonal antibody (anti-AGR2, FOLR1, GLY, and/or SMRP) solution were dripped onto the surface of 6-MHA and EDC/NHS-activated HSPEs and incubated for 30 min. Then, 5 µL (single) and 3 µL (quadruple) of 1% BSA solution were dropped onto the surface of HSPEs and incubated for 30 min. Finally, 5 µL (single) and 3 µL (quadruple) of target antigen (AGR2, FOLR1, GLY, and/or SMRP) solution were dropped onto the surface of HSPEs and incubated for 30 min. Schematic demonstration of the preparation steps of single- and panel immunosensors and photographs are given in Fig. 1C-E.

## Results and discussion

### Morphological, chemical, and electrochemical characterizations of the electrodes

#### Hand-made electrode characterization

**Fig. 2:**
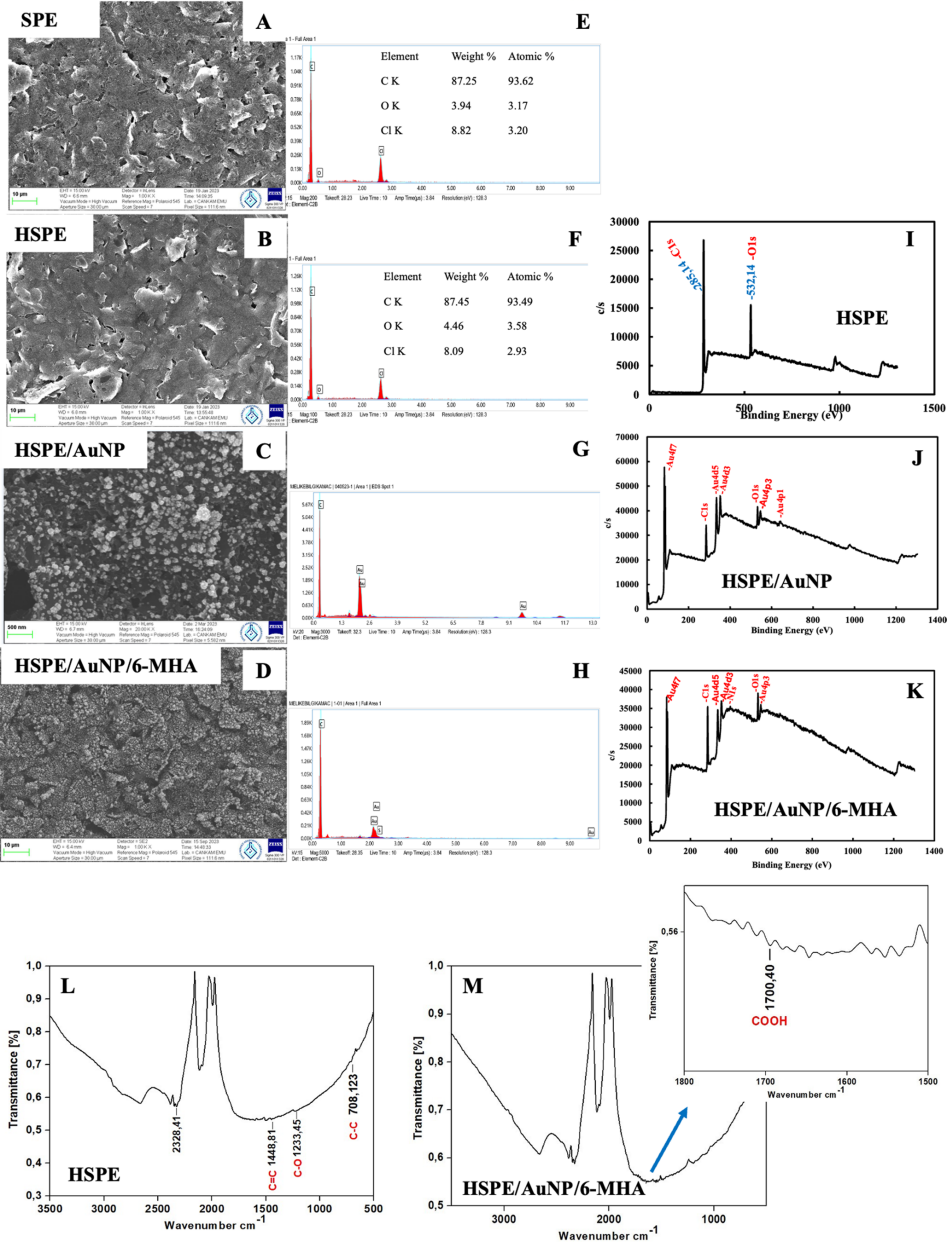
SEM images **(A-D)**, EDX analysis **(E-H)**, XPS analysis **(I-K)** of HSPE, HSPE/AuNP, and HSPE/AuNP/6-MHA, FT-IR analysis **(L-M)** of the HSPE and HSPE/AuNP/6-MHA

Morphological, chemical, and electrochemical characterizations of the produced single HSPEs were performed, and the results were compared with SPEs. SEM images for morphological characterization reveal that HSPEs exhibit a rougher structure than SPEs (Fig. 2A-B). According to the EDX analysis results (Fig. 2E-F), the elemental analysis distributions of SPEs and HSPEs are seen to be close to each other [44]. FT-IR and XPS analyses were performed for the chemical characterization of the HSPEs produced and are given in Fig. 2I and L. In the FT-IR spectrum (Fig. 2L), the peak at 732 cm⁻¹ originates from C-C stretching vibrations, the peak at 1450 cm⁻¹ originates from C = C stretching vibrations, and the peak at 2328 cm⁻¹ originates from the vibrations of alkyne groups. The peaks at 1237 cm⁻¹ and 1017 cm⁻¹ originate from the vibrations in the C-O bond in the epoxy and alkoxy functional groups, respectively [45]. In the XPS analysis, the C1s peak (Fig. 2I) originates from unoxidized C and carbon atoms in C-O, C = O, C-O-C, and H-O-C = O compounds on the surface. The peak at 285.14 eV in the XPS spectrum indicates the presence of unoxidized carbon and C-O, C = O, C-O-C, and H-O-C = O groups on the electrode surface. Peaks for unoxidized carbon (C) at 284.5 eV, C-O at ~ 286.5 eV, epoxy/ether group (C-O-C) at ~ 288 eV, and carbonyl carbon, carboxylate (H-O-C = O) carbon at ~ 286 eV have been reported in the literature [46]. In addition, the appearance of the O1s peak at 532.14 eV proves the presence of C-O, C = O, and H-O-C = O groups on the electrode surface [46].

**Fig. 3:**
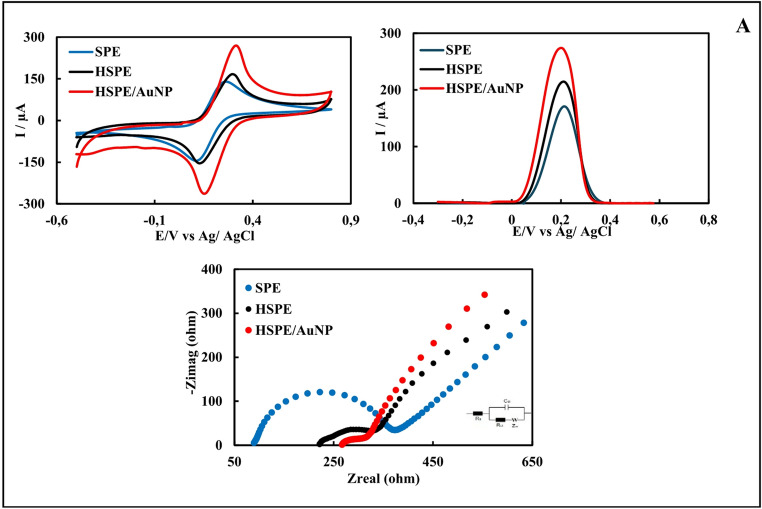

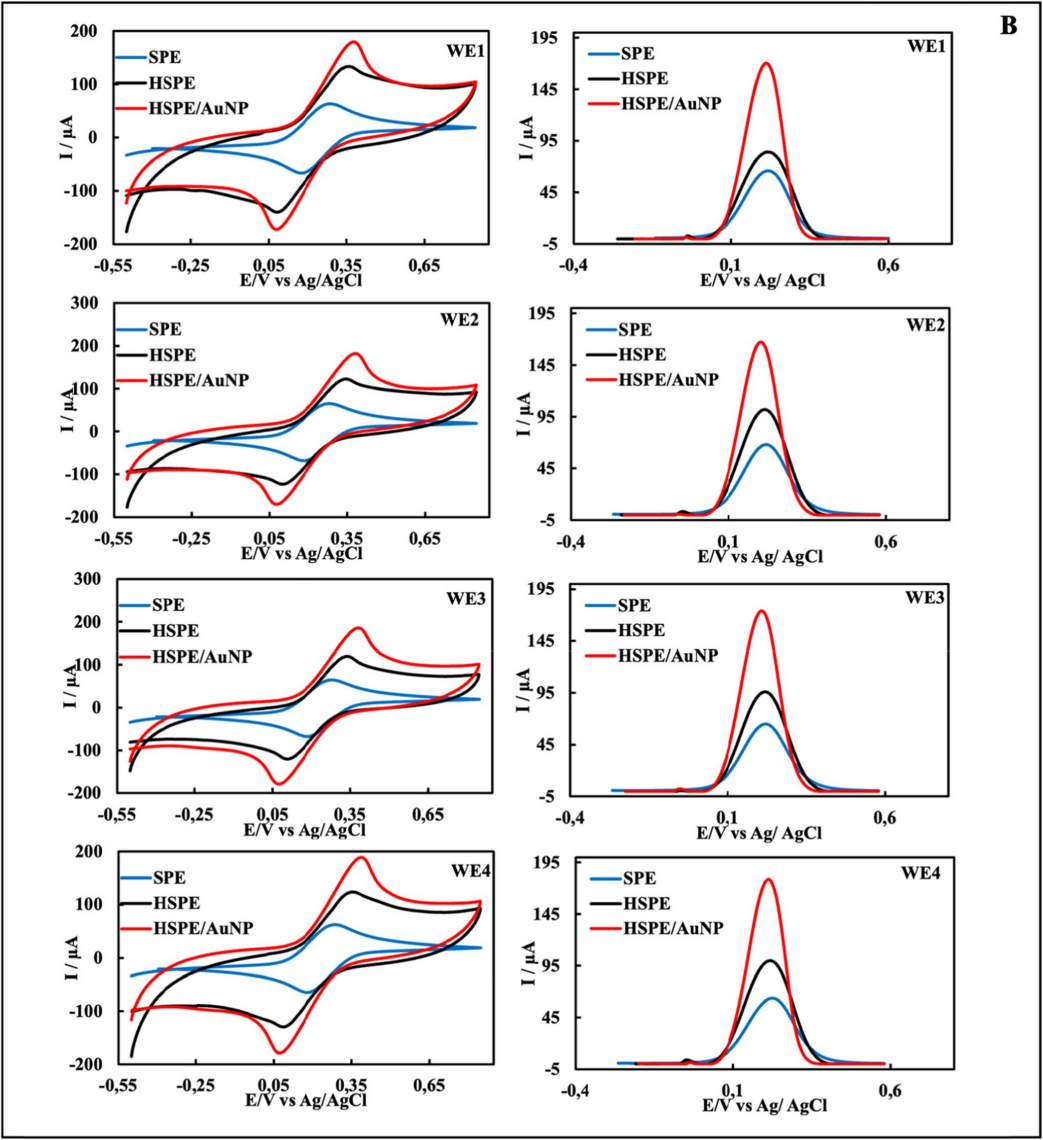
CVs, DPVs, and EISs of single SPE, HSPE, and HSPE/AuNP **(A)**; CVs and DPVs of quadruple SPE, HSPE, and HSPE/AuNP **(B)**

To compare the electrochemical performance of single and quadruple HSPEs with commercial SPEs, EIS (single electrodes only), CV, and DPV methods were applied (Fig. 3) in redox probe solution. Ten different electrodes were used for each electrode type, and measurements were performed in triplicate. Analyses of single and quadruple electrodes revealed that the average peak current values ​​(Ipa_avg_) of HSPEs were significantly higher than those of SPEs. A high Ipa_avg_ is generally associated with a faster electron transfer at the electrode surface or a larger active surface area [47]. These results were confirmed by taking the CVs of single SPE and single HSPEs in the redox probe solution at different scan speeds and then calculating their electroactive surface areas (see Supplementary Files, Sect. 2.1, and Figure S1A-D). Analyses of single electrodes also found that HSPEs had lower average charge transfer resistance values ​​(Rct_avg_) than SPEs (Table S1). A lower Rct_avg_ value indicates that the electron transfer kinetics between the redox probe solution and the electrode surface are faster, and electrochemical reactions occur more easily. These results support the conclusion that the electrode has higher conductivity [47]. Using the electrochemical characterization results of HSPEs and SPEs, t-tests were performed at the 95% confidence level to determine whether the differences between Ipa_avg_ and Rct_avg_ values ​​were statistically significant (t < 0.05). Statistical analyses confirmed that these performance differences between the HSPE and SPE electrodes were statistically significant (Table S1). This statistical validity demonstrates that the observed performance superiority is not due to mere random variation but rather represents a true improvement due to differences in the electrode fabrication method. All findings indicate that the produced HSPEs are more conductive than commercial SPEs. Additionally, an approximate cost calculation was performed, and the unit cost of commercial SPEs was determined to be approximately $4.28, while the unit cost of HSPEs was approximately $0.35. These results indicate that the handmade electrodes produced in our study outperformed commercial electrodes in terms of both cost and electrochemical performance.

#### AuNP modified electrodes

To form a sufficient amount of AuNPs to give a significant electrochemical response on HSPE surfaces, optimization studies were carried out for HAuCl₄ solution solvent type (pH 7.0 PBS (non-acidic) and 0.5 M H₂SO₄ pH 7.0 PBS (acidic)), buffer solution pH (pH 6, 7, and 8), concentration (0.6 mM, 4 mM, 6 mM, 60 mM, and 600 mM HAuCl₄), electrochemical method cycle number (5, 10, 15, and 20 cycles), and scan rate (25, 50, and 100 mV s⁻¹). To determine the optimum conditions, voltammograms and electrochemical impedance spectra were evaluated for electrochemical characterization, while SEM images and EDX analysis results were evaluated for morphological characterization. Based on the analysis results, the optimum HAuCl₄ concentration was determined as 4 mM, the solution system was a pH 7.0 PBS buffer, the scan rate was 50 mV/s, and the electrochemical method cycle number was 10 n. Detailed optimization studies are provided in the Supplementary File.

According to the EIS (only single electrodes), CV, and DPV given in Fig. 3, the modification of AuNPs on HSPEs was successfully carried out. In voltammograms, the Ipa_avg_ values ​​of single and quadruple HSPE/AuNPs increased compared to the Ipa_avg_ value of HSPEs. The Rct_avg_ value of HSPE/AuNPs decreased significantly in EIS. These results indicate that AuNPs increased the conductivity and electrochemical activity of the electrode. SEM-EDX and XPS images of HSPE/AuNP prepared under optimum conditions are given in Fig. 2C and G, and 2J. The presence of spherical AuNPs in the SEM images, the presence of the Au element in the EDX spectrum, and the presence of Au4f7, Au4d5, and Au4d3 peaks in the XPS spectrum prove the modification of AuNPs on HSPEs.

Two different sulfur compounds, 3-MPA and 6-MHA, were tested to form SAM films on HSPE/AuNP prepared under optimum conditions. Optimization studies were conducted for concentration and incubation times to determine the sulfur compound that best formed the SAM layer. Voltammograms and electrochemical impedance spectra obtained during electrochemical characterization were evaluated to determine the optimum conditions. As a result of the studies, the best-performing sulfur compound was determined to be 6-MHA, with an optimum concentration of 100 mM and an incubation time of 18 h. Details of the optimization studies are provided in the Supplementary File (Sect. 2.1.2).

SAM films of 6-MHA were formed on HSPE/AuNP under optimum conditions (18 h, 100 mM), and SEM-EDX, XPS, and FT-IR analyses were performed (Fig. 2D, H and K, and 2M). SEM images clearly show that the surface of the AuNP/6-MHA modified electrode has a dense and rough structure. These images provide evidence that 6-MHA molecules successfully formed SAM layers on the AuNP surfaces. According to EDX analysis results, the presence of sulfur on the surface provides further evidence of SAM layers [48]. The absorbance band observed at 1701.40 cm⁻^1^ in the FT-IR spectrum is the carbonyl (C = O) stretching vibration, confirming the presence of 6-MHA molecules (Fig. 2M). However, the absence of the expected S-H stretching vibration at 2561 cm⁻¹ suggests that the -SH group interacts with the gold surfaces to form Au-S bonds, thus forming the SAM structure [49]. In the XPS spectrum in Fig. 2K, the S 2p3/2 signal at 162.5 eV is characteristic of thiolate (Au-S) bonds and provides definitive evidence that 6-MHA molecules are covalently bound to gold surfaces [49]. This result agrees with the FT-IR data and confirms that the modification process was successful.

In the preparation of immunosensors, the surfaces of HSPE/AuNP/6-MHA electrodes were first activated with EDC-NHS crosslinkers for the immobilization of the antibody onto the surface. Since carboxylic acid (-COOH) groups on the surface of HSPE/AuNP/6-MHAs cannot directly bind to the primary amine (-NH_2_) groups in the structure of antibodies, EDC-NHS, a common and effective crosslinker that provides covalent bonding of these two groups, was used [43, 44, 50, 51]. To prove the presence of EDC-NHS on the surface of the electrodes, SEM, FT-IR, and XPS analyses of HSPE-AuNP-6-MHA/EDC-NHS were performed (Figure S10 A-D). According to the spectra and images, the presence of EDC-NHS on the electrode surface was proven (Detailed descriptions and figures are provided in the Supplementary File, Sect. 2.2).

### Electrochemical characterizations of single and panel immunosensors

**Fig. 4:**
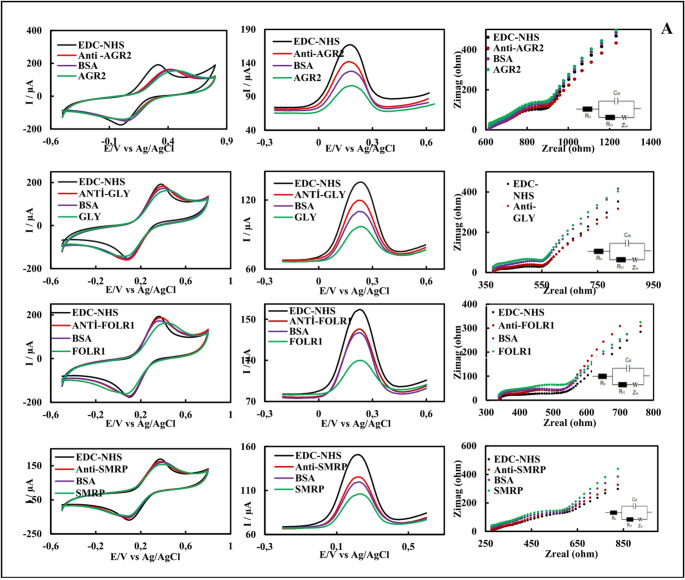

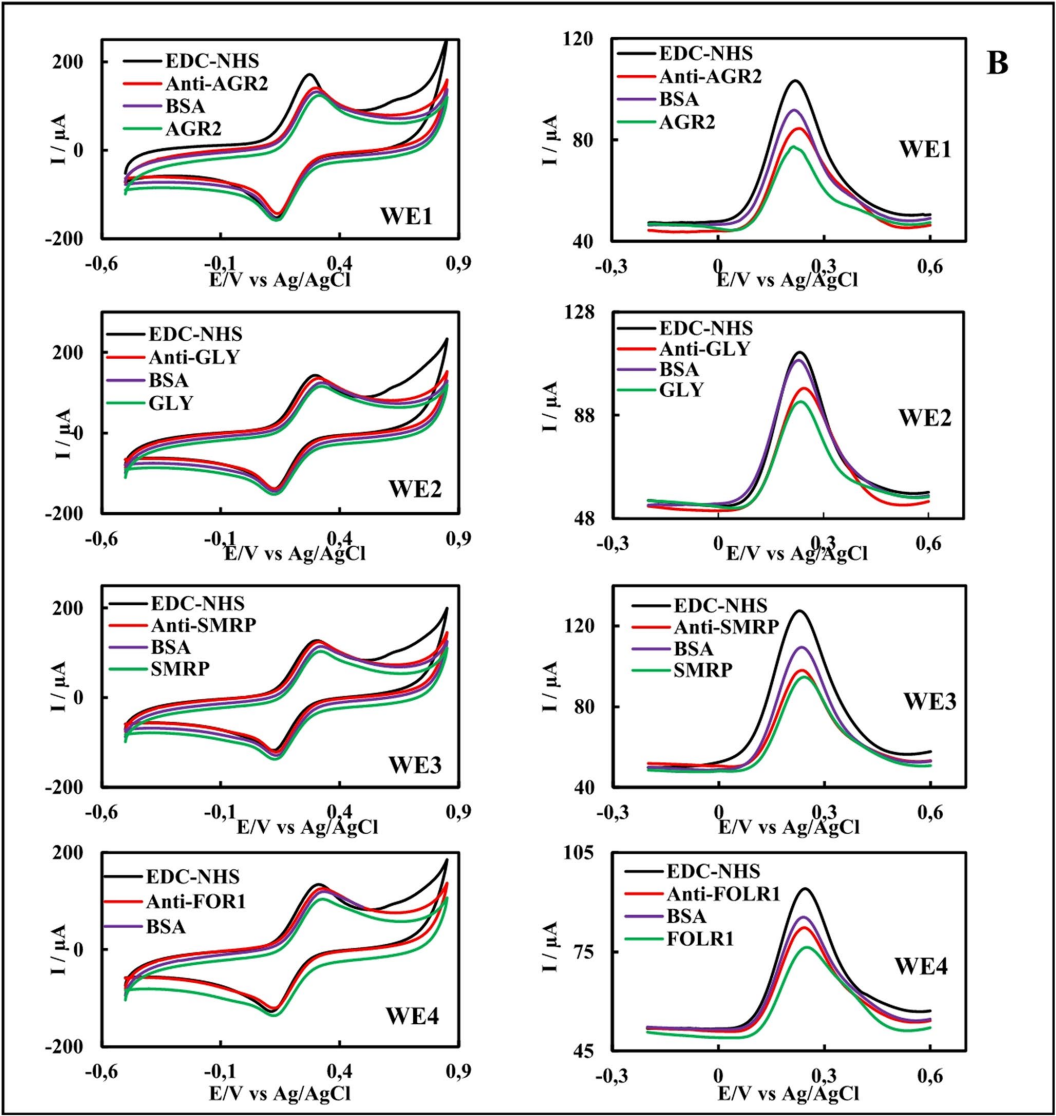
Electrochemical characterization of the single **(A)** and panel **(B)** AGR2, GLY, FOLR1, and SMRP immunosensors

The EIS (only for single immunosensors), CV, and DPV of single immunosensors and panel immunosensors prepared with HSPEs are shown in Figs. 4A and B, and the Ipa_avg_, Epa_avg_, and Rct_avg_ values ​​are shown in Table S10. In the voltammograms of the single-mode immunosensors and panel immunosensors, a decrease in the Ipa_avg_ values ​​was observed during the transition from the EDC-NHS step to the antibody incubation step. An increase in the Rct_avg_ value was observed in the EISs (only for single immunosensors). Diffusion decreased as the antibody formed an insulating layer on the surface. These results demonstrate that the antibody successfully bound to the surface [43]. The insulating layer increased further after BSA and antigen incubations. As a result, the Ipa_avg_ values ​​decreased, and the Rct_avg_ values ​​increased. According to the electrochemical characterization results, single and panel AGR2, FOLR1, GLY, and SMRP immunosensors were successfully prepared.

### Optimization studies of immunosensors

**Fig. 5:**
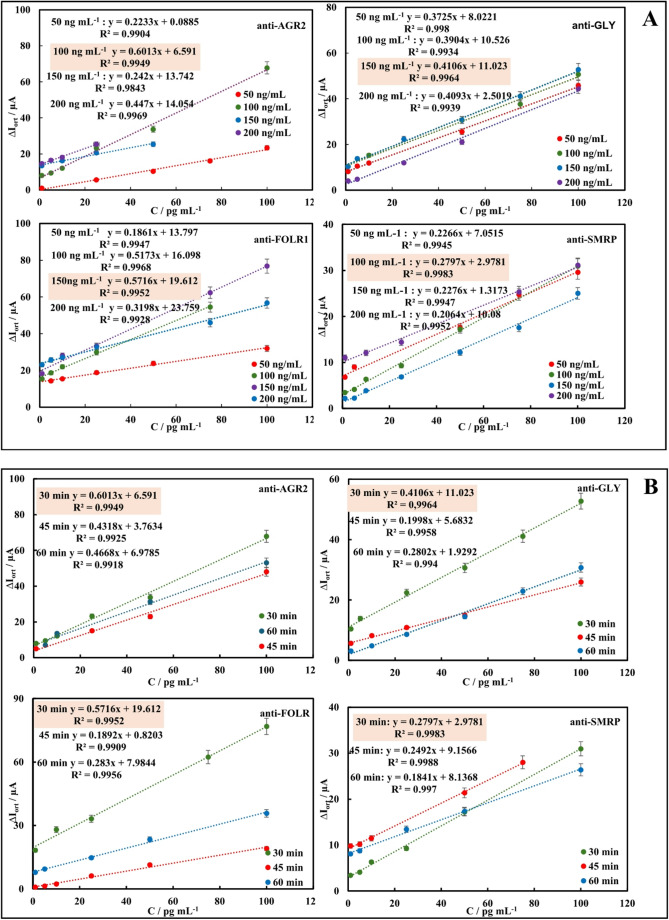

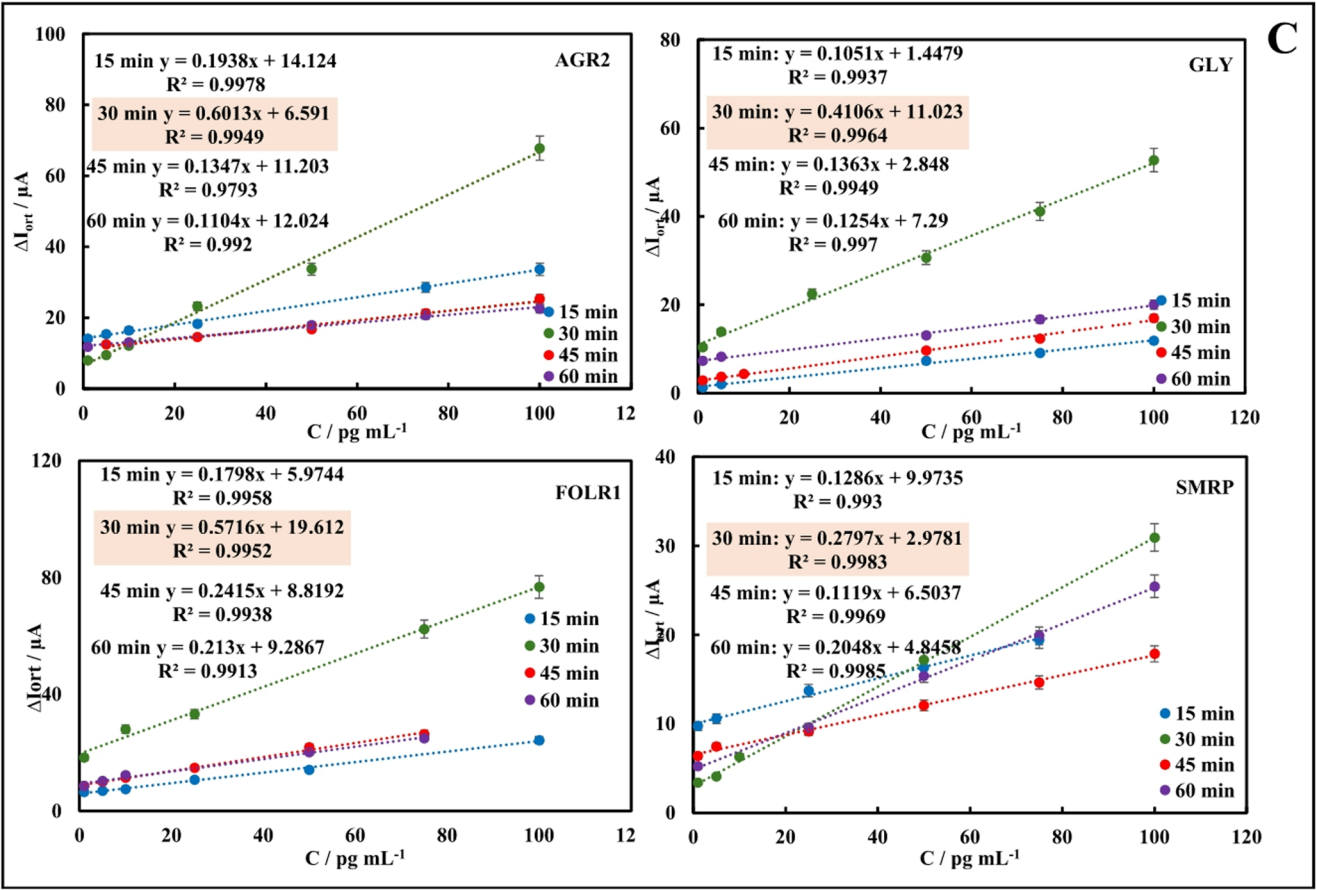
Optimization studies of the single AGR2, GLY, SMRP, FOLR immunosensors; antibody concentration **(A)**, antibody incubation time **(B)**, and antigen incubation time **(C)**

Optimization studies are necessary and critical for the optimal performance of immunosensing systems. Therefore, the key steps in immunosensor preparation—antibody concentration, antibody, and antigen incubation times—have been optimized for single immunosensors. Optimum parameters were applied to panel immunosensor systems. Immunosensors prepared for optimization studies were incubated with target antigens. Target antigen analyses were performed using DPV in a redox probe solution. Plots of peak current differences (ΔI = I_antigen_ - I_BSA_) versus target antigen concentrations were plotted for each immunosensor and are presented in Figs. 5A-C.

In antibody concentration optimization studies, concentrations of 50 ng mL^− 1^, 100 ng mL^− 1^, 150 ng mL^− 1^, and 200 ng mL^− 1^ were tested for each antibody. Considering the slope and correlation coefficients of the graphs in Fig. 5A, the optimum concentrations for anti-AGR2, anti-FOLR1, anti-GLY, and anti-SMPR were determined to be 100 ng mL^− 1^, 150 ng mL^− 1^, 150 ng mL^− 1^, and 100 ng mL^− 1^, respectively. In optimization studies of the incubation times of each antibody, incubation times of 30, 45, and 60 min were tested. Considering the slope and correlation coefficients of the graphs in Fig. 5B, the optimum incubation times of anti-AGR2, anti-FOLR1, anti-GLY, and anti-SMPR were determined as 30 min each. In optimization studies of the incubation times of target antigens, incubation times of 15, 30, 45, and 60 min were tested. Considering the slope and correlation coefficients of the graphs in Fig. 5C, the optimum incubation times of AGR2, FOLR1, GLY, and SMPR were determined as 30 min each.

### Analytical performance of the single and panel immunosensors

**Fig. 6:**
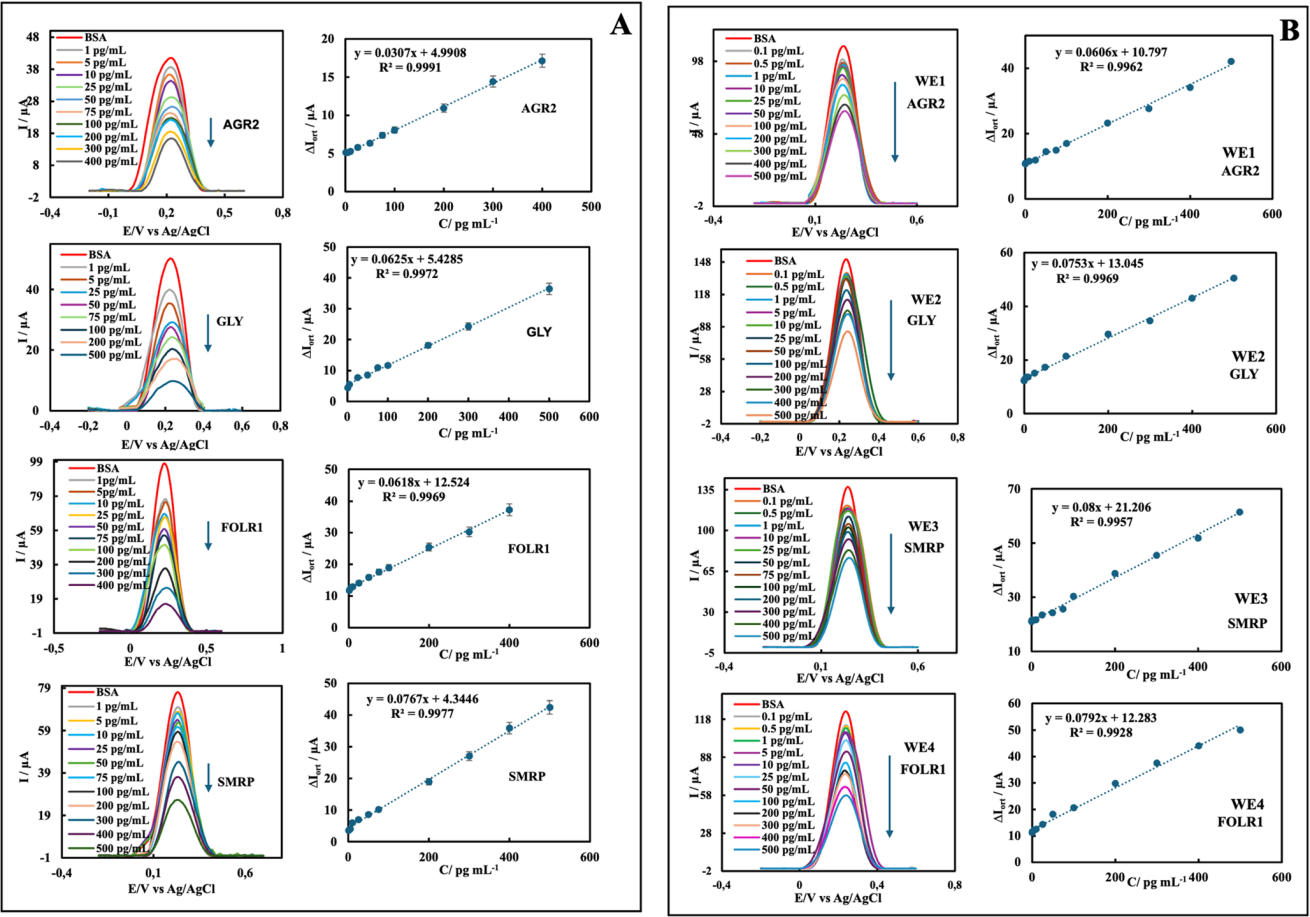
Individual and simultaneous determination of AGR2, GLY, SMRP, and FOLR1 at different concentrations: DPVs and calibration plots of **(A)** single immunosensors, **(B)** panel immunosensors

To determine the linear detection ranges of single and panel immunosensors, analyses of target antigens at different concentrations using immunosensors were carried out individually and simultaneously with DPV measurements in the redox probe solution. The voltammograms recorded during the analysis and the graphs of the target antigens at different concentrations against the ∆Ipa_avg_ values ​​obtained from the voltammograms are given in Fig. 6A-B. Limits of detection (LOD), sensitivity, and linear detection ranges calculated based on regression analysis results performed in Excel are given in Table S11. These results show that both single and panel immunosensors respond to target antigens with a wide linear range, low LOD, and high reproducibility. In addition, panel immunosensors can rapidly and conveniently detect four target antigens simultaneously. In the comprehensive literature review (Table 1), it was seen that panel electrochemical immunosensor systems that can simultaneously determine the biomarkers AGR2, FOLR1, GLY, and SMRP have not been previously proposed. Reported studies have generally focused on single immunosensor systems targeting only one of these biomarkers, and the number of these studies is limited. According to our study in Table 1, expensive electrodes such as Au were used in the AGR2 immunosensor study [15], which provided a lower detection limit, and FET systems were used in the FOLR1 immunosensor study [23]. However, these immunosensor platforms have disadvantages, including complex manufacturing processes, high-cost semiconductor materials, and limited scalability. In contrast, the HSPE/AuNP-based system developed in this study offers a low-cost, portable solution with a much simpler preparation procedure; in this respect, it provides a more accessible platform for clinical applications. Reference levels for serum biomarkers AGR2, GLY, SMRP, and FOLR1 in healthy individuals are approximately 163.67 ± 50.38 ng/ml, 5.7 ± 1.6 ng/ml, 0.05–0.4 nM, 327–693 pg/ml, and 0.8–2.5 ng/mL, respectively [52–55]. The LODs of the HSPE/AuNP-based immunosensors developed in our study were above the fg/mL levels achieved in some studies in the literature. However, the obtained LOD values ​​are well below the reference concentration ranges of these biomarkers in the serum of healthy individuals. This shows that the immunosensors we produce can reliably measure the physiological and pathological levels necessary for clinical diagnosis, even at levels lower than those required.

**Table 1:** Performance comparisons of individual AGR2, GLY, SMRP*, and FOLR1 immunosensors in the literature and the performance comparisons of the immunosensors developed in our study

	LOD	Linear Range	Method	Ref.
Au/4-ATP/GA/anti-AGR2/BSA/AGR2	0.000093 pg mL^− 1^	0.0001–0.1.0001.1 pg mL^− 1^	EIS	[15]
HSPE/AuNP/Anti-AGR2/BSA/AGR2	0.309 pg mL^− 1^	1–400pg mL^− 1^	DPV	This work
GDE/Cys/Ghy/ant-GLY/BSA/GLY	430 pg mL^− 1^	1–10^6^ pg mL^− 1^	SWV	[19]
HSPE/AuNP/Anti-GLY/BSA/GLY	0.360 pg mL^− 1^	1–500 pg mL^− 1^	DPV	This work
FET/MoS_2_	0.057 pg mL^− 1^	0.1 pg mL^− 1^ − 10,000 pg mL^− 1^	EIS	[24]
ITO/[PAH/FA]_3_/Anti-FOLR1/BSA/FOLR1	700 pM	10,000–40,000 pM		[25]
HSPE/AuNP/Anti-FOLR1/BSA/FOLR1	0.307 pg mL^− 1^	1–400 pg mL^− 1^	DPV	This work
HSPE/AuNP/Anti-SMRP/BSA/SMRP	0.334 pg mL^− 1^	1–400 pg mL^− 1^	DPV	This work

*SMRP was analyzed only in the present biosensor system. The previously reported biosensors summarized in this table did not include SMRP detection; therefore, it is not listed for comparison

The hand-made SPE-based panel immunosensor system that can simultaneously determine four biomarkers produced in this study is a first in the literature. Repeatability and reproducibility studies of the prepared single and panel immunosensors were performed. Good repeatability was observed for single immunosensors up to 7 replicates and for panel immunosensors up to 6 replicates; reproducibility of single and panel immunosensors was good for up to 6 electrodes; and %RSD values ​​were below 5% (Table S12).

**Fig. 7:**
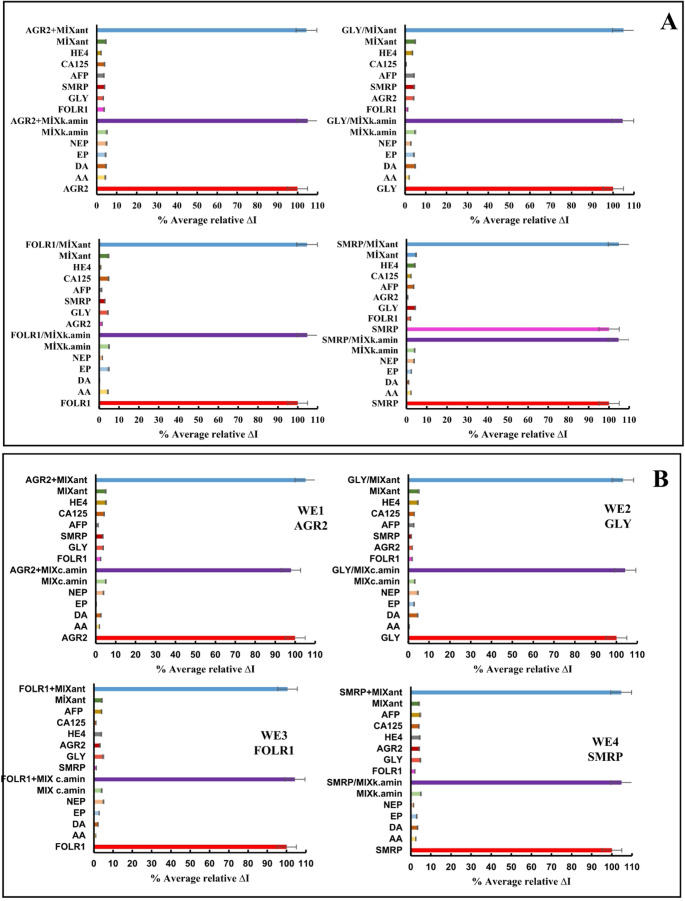

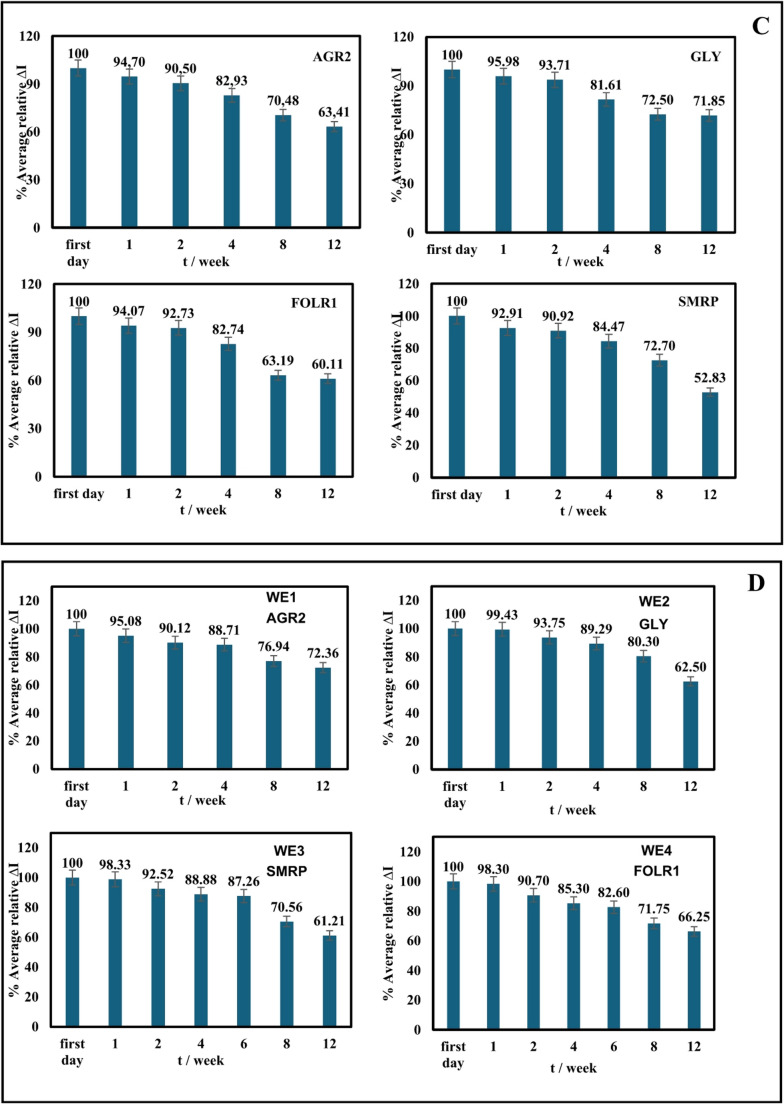
Selectivity study **(A-B)** and shelf-life study **(C-D)** of single and panel immunosensors

For the selectivity study of the produced single and panel immunosensors, the %average relative ∆I values ​​were calculated from individual and simultaneous DPV measurements of the immunosensors incubated with the target antigen. Similarly, %average relative ∆I values ​​were calculated using the DPV measurements of single and panel immunosensors incubated with antigens other than the target antigen (AGR2, SMRP, FOLR1, GLY, HE4, CA125, and AFP) individually, their mixtures, and mixtures of all antigens, including the target antigen. Selectivity test results obtained for single and panel immunosensors are plotted in Fig. 7A-B. According to the plots, analyses performed with both single and panel immunosensors show that selectivity is acceptable (error below 5%) in the presence of antigens other than the target antigen and other electroactive substances. These results provide strong evidence that antibodies immobilized on the surfaces of single and panel AGR2, GLY, FOLR1, and SMRP immunosensors bind to their target antigens with high affinity and specificity. They also demonstrate that other electroactive compounds have no inhibitory or disruptive effects on the antibody-antigen interaction, and that the antibody binding sites are highly specific and are not blocked by these small molecules. The acceptable levels of interference values ​​demonstrate the potential for reliable operation of our immunosensors, even in complex matrices such as real biological samples.

To test the shelf life of the prepared single and panel immunosensors, immunosensors were prepared up to the BSA step and stored dry in petri dishes in an incubator set to + 4 °C.

The shelf life of the developed single and panel immunosensors was tested individually and simultaneously using DPV measurements for 12 weeks. The immunosensors’ responses to their target antigens were tested on the first day of preparation and then at storage periods of 1, 2, 4, 8, and 12 weeks, respectively. As seen in Figs. 7C-D, their responses to their target antigens were good even after 12 weeks of storage.

### Analysis of target antigens in commercial blood serum with immunosensors and ELISA kits

Individual and simultaneous analyses of target antigens in commercial human blood serum (Sigma H6914) were performed by DPV measurements using prepared single and panel immunosensors. Serum samples were diluted 1:100 with pH 7.4 PBS. The accuracies of the analysis results of the immunosensors prepared for each target antigen were compared with the analyses performed with the reference method, ELISA kits. Since the detection ranges of the ELISA kits were different (for AGR2: 1.56–100 ng mL⁻¹, for GLY: 0.78–50.78 ng mL⁻¹, for FOLR1: 46.9–3000 pg mL⁻¹, for SMRP: 62.5–4000 pg mL⁻¹), sample preparation procedures were also different. Serum samples containing AGR2 and GLY were prepared by adding AGR2 and GLY to the diluted serum samples at three different concentrations (10 ng mL⁻¹, 20 ng mL⁻¹, and 30 ng mL⁻¹). Then, the serum samples were diluted until they reached the detection range of the relevant immunosensors (100 pg mL⁻¹, 200 pg mL⁻¹, and 300 pg mL⁻¹). Serum samples containing FOLR1 and SMRP were prepared by adding FOLR1 and SMRP antigens to the diluted serum sample at different concentrations (100 pg mL⁻¹, 200 pg mL⁻¹, and 300 pg mL⁻¹) by the standard addition method. Serum samples containing target antigens were incubated with the developed single and panel immunosensors, and then separate and simultaneous electrochemical analyses were performed in triplicate. In the analyses performed with single and panel immunosensors, the % errors calculated using Ipa_avg_ values ​​were found to be less than 5%, and the % recoveries are between 97.9% and 103.72% for single immunosensors and 95.32% and 103.61% for panel immunosensors (Table S13).

AGR2, GLY, SMRP, and FOLR1 levels were determined in commercial human serum samples using ELISA kits. The concentrations of target antigens in commercial human serum using ELISA kits were calculated using absorbance data measured in UV/VIS and calibration graphs of the ELISA kits. The results of antigen analyses performed with ELISA kits and prepared single and panel immunosensors were compared statistically using the F-test and t-test, and percentage difference values ​​were also calculated (Tables S14-S15). According to Table S14, the fact that the calculated F values ​​at all concentrations are less than the critical value of F indicates that there is no statistically significant difference (i.e., the variances are homogeneous) between the variances of the data obtained from ELISA and single/panel immunosensors. According to Table S14, for the t-test, the p-values ​​are less than 0.05, and the t-values ​​calculated for panel and single immunosensors at all concentrations are less than the t-_critical_. These results show that the target antigen analysis results obtained with single and panel immunosensors are statistically consistent with the results obtained with ELISA, and there is no significant difference between them. The accuracy of the single and panel immunosensors we produce has been verified by ELISA.

## Conclusion

Single and quadruple HSPEs, offering superior performance and lower cost compared to commercial SPEs, were modified with AuNP and demonstrated high sensitivity over a wide linear range. Surface analyses (SEM, XPS, FTIR) confirmed the robust sensor fabrication. For the first time in this study, an electrochemical panel immunosensor system was prepared using HSPEs for the simultaneous determination of potential ovarian cancer biomarkers AGR2, GLY, FOLR1, and SMRP. Individual and simultaneous analyses of target antigens were performed using the prepared immunosensors, yielding good reproducibility, high accuracy, and low detection limits of pg mL^− 1^ over a wide linear range. In all selectivity studies, the percentage of errors was below 5%, demonstrating that the immunosensor platforms are highly selective towards their target antigens and can be reliably used in complex biological environments. The fact that the immunosensors exhibited a good response to their target antigens even after 12 weeks of storage demonstrates the potential for mass production and clinical use of this low-cost platform. The clinical validity of the multi-platform was validated by recovery rates of over 95% in human serum samples and by ELISA.

In conclusion, this study represents an innovative contribution to the field by presenting, for the first time in the literature, an electrochemical panel system that enables the simultaneous determination of AGR2, GLY, FOLR1, and SMRP biomarkers with high selectivity and accuracy, and also offers a low-cost, reproducible, replicable, and clinically compatible platform. In the future, validation of electrochemical panel systems in clinical samples will enable them to become important diagnostic tools for the early detection of other tumors and cancers, particularly ovarian tumors.

## Supplementary Information

Below is the link to the electronic supplementary material.

Supplementary Material 1 (DOCX 42.2 MB)

## Data Availability

Data is provided within the manuscript or supplementary information files.
