# Supplementary material for: Disposable electrochemical panel immunosensing systems for the simultaneous detection of potential biomarkers of ovarian cancer
Source: Mikrochim Acta. 2026 Jan 10;193(2):83. doi: 10.1007/s00604-025-07820-8 (PMC12790523; doi:10.1007/s00604-025-07820-8)
Supplement: Supplementary file 1 — Supplementary Material 1 (DOCX 42.2 MB) [file 604_2025_7820_MOESM1_ESM.docx]

**Supplementary File**

**Disposable Electrochemical Panel Immunosensing Systems for the Simultaneous Detection of Potential Biomarkers of Ovarian Cancer**

Melike BİLGİ KAMAÇ^1*^, Ayşenur YILMAZ KABACA^2^, Merve YILMAZ ÇILÇAR^1^, Muhammed ALTUN^3^, Mustafa Kemal SEZGİNTÜRK^4*^,

^1^Çankırı Karatekin University, Faculty of Science, Chemistry Department, Çankırı, 18100, Türkiye

^2^Çankırı Karatekin University, Şabanözü Vocational School, Department of Medical Laboratory Techniques, Çankırı, 18000, Türkiye

^3^Çankırı Karatekin University, Eldivan Vocational School of Health Services, Department of First and Emergency, Çankırı, 18000, Türkiye

^4^Çanakkale Onsekiz Mart University, Faculty of Engineering, Bioengineering Department, Çanakkale, 17000, Türkiye

***Corresponding author: e-mail: [melikesahin@karatekin.edu.tr](mailto:melikesahin@karatekin.edu.tr), [msezginturk@comu.edu.tr](mailto:msezginturk@comu.edu.tr)

**1. Experimental**

**1.1. Chemicals**

Chloroauric acid (HAuCl_4_), bovine serum albumin (BSA), potassium hexacyanoferrate (K_3_Fe(CN)_6_), potassium hexacyanoferrite (K_4_Fe(CN)_6_, N-(3-dimethylamino propyl)-N′-ethyl carbodiimide (EDC), N-hydroxysuccinimide (NHS), human serum (from male AB clotted whole blood, H6914), 6-mercapto hexanol (6-MHA), 3 mercaptopropionic acid (3-MPA), Silver paste (Ag) and silver-silver chloride (Ag/AgCl) were obtained from Sigma-Aldrich (USA). Carbon ink was obtained from Dycotech. Polyethylene terephthalate (PET) was obtained from the public market. Recombinant human folate receptor 1 (FOLR1) protein and anti-folate receptor I (Anti-FOLR1) were purchased from RD System. Anti-anterior gradient-2 protein (Anti-AGR2) was purchased from Abnova. Recombinant human cancer antigen 125 (CA125/MUC16), alpha-fetoprotein (AFP), human epididymis protein 4 (HE4), glycodelin (GLY), soluble mesothelin-related protein (SMRP), AGR2 proteins, and anti-SMRP were purchased from Novus Biologicals USA. ELISA kits coded MBS2024128 for AGR2, MBS704902 for GLY, MBS177335 for FOLR1, and MBS702446 for SMRP were obtained from My BioSource. Ethanol (EtOH), potassium chloride (KCl), potassium dihydrogen phosphate (KH_2_PO_4_), potassium hydrogen phosphate (K_2_HPO_4_), and hydrochloric acid (HCl) were obtained from Merck. To make a phosphate buffer solution (PBS), K_2_HPO_4_, KH_2_PO_4_, and KCl were utilized. All of the solutions used were prepared with ultrapure water (Millipore,18MΩ cm).

**1.2. Instruments**

The voltammetric analysis was performed using a DropSens µStat 4000P Multi Potentiostat from Methrom DropSens (Oviedo, Spain) and operated by a PC running DropView 800 software. Gamry Potentiostate/Galvanostate, Reference 1010E (Gamry Instruments, Warminster, USA), electrochemical impedance spectroscopy (EIS) measurements were performed on a computer running EChem Analyst. The commercial SPEs were purchased by Methrom DropSens (Oviedo, Spain, DRX110/DPR-4W110). Single SPEs consist of a combined three-electrode system with a working (WE) and a counter electrode of carbon and a reference electrode of silver. Quadrupole SPEs consist of a combined three-electrode system with a four WE and a carbon counter electrode of carbon and a reference electrode of silver. For chemical and morphological characterizations, Perkin Elmer Fourier transform infrared (FT-IR), ZEISS Gemini 1 Field Emission Scanning Electron Microscope (FE-SEM), and Panalytical Empyrean X-ray Photoelectron Spectroscopy (XPS) devices were used, respectively. A cutter printer (Silhouette Cameo4) was used to create the pattern of the electrodes. ELISA absorbance values ​​were measured using a microplate reader device (BioTek ELx800) operating at a wavelength of 450 nm.

**1.3. Electrochemical measurements**

Electrochemical characterizations were carried out with cyclic voltammetry (CV), differential pulse voltammetry (DPV), and EIS in redox probe solution (5 mM redox probe solution: K_3_Fe(CN)_6_/K_4_Fe(CN)_6_ in 1 M KCl). EIS measurements were carried out at a 50.000 Hz - 0.05 Hz frequency range. The following parameters were used for CV measurements: potential ranges of -0.5V to +0.8V at 50 mV s^-1^. DPV measurement parameters were pulse potential 70 mV, pulse time 0.1 s, step potential 5 mV, and scan rate 5 mV s^-1^. Optimum operating parameters (antibody concentration, antibody, and antigen incubation times) and analytical characterizations (linear range, repeatability, reproducibility, stability, etc.) of the single and panel immunosensors were performed by the DPV method. Measurement of target antigen levels in commercial blood serum by the DPV method.

**1.4 Production of handmade electrodes**

The pattern of single and quadruple electrodes was created using Silhouette Studio software and then transferred onto an adhesive vinyl template. The vinyl stencil, removed from the cutter printer, was glued to the PET. Carbon ink was poured onto the vinyl stencil and then applied across the stencil with a squeegee. The stencil was then left in an oven at 60°C for 30 minutes to dry. In the next step, Ag/AgCl ink was applied to the reference electrode section with a fine brush, and Ag ink was applied to the connection parts of the electrode; then, curing was carried out at 60°C for 30 minutes. The vinyl template was carefully removed from the PET surface using forceps. The lamination template prepared with the Silhouette Studio cutting device was carefully pasted onto the PET surface. Then, a heat-press process was applied at 100 °C for 10 seconds, and the electrodes were cut and made ready for use.

**2. Results and Discussion**

**2.1. Electrochemical characterizations of handmade electrodes**

CV was applied to single SPEs and HSPEs in the redox probe solution at different scan rates, and the anodic peak currents were plotted against the square root of the scan rate (Figure S1 A-D). Using the plots and the Randles-Sevcik equation (Ip=2.69x105n3/2AeaC√Dⱱ), the electroactive surface areas (Aea) of SPEs and HSPEs were calculated [1]. The Aea of ​​ SPEs was calculated as 0.1252 cm², and the Aea of ​​ HSPE was calculated as 0.2690 cm². These results prove that HSPEs have better electrochemical performance than SPEs.


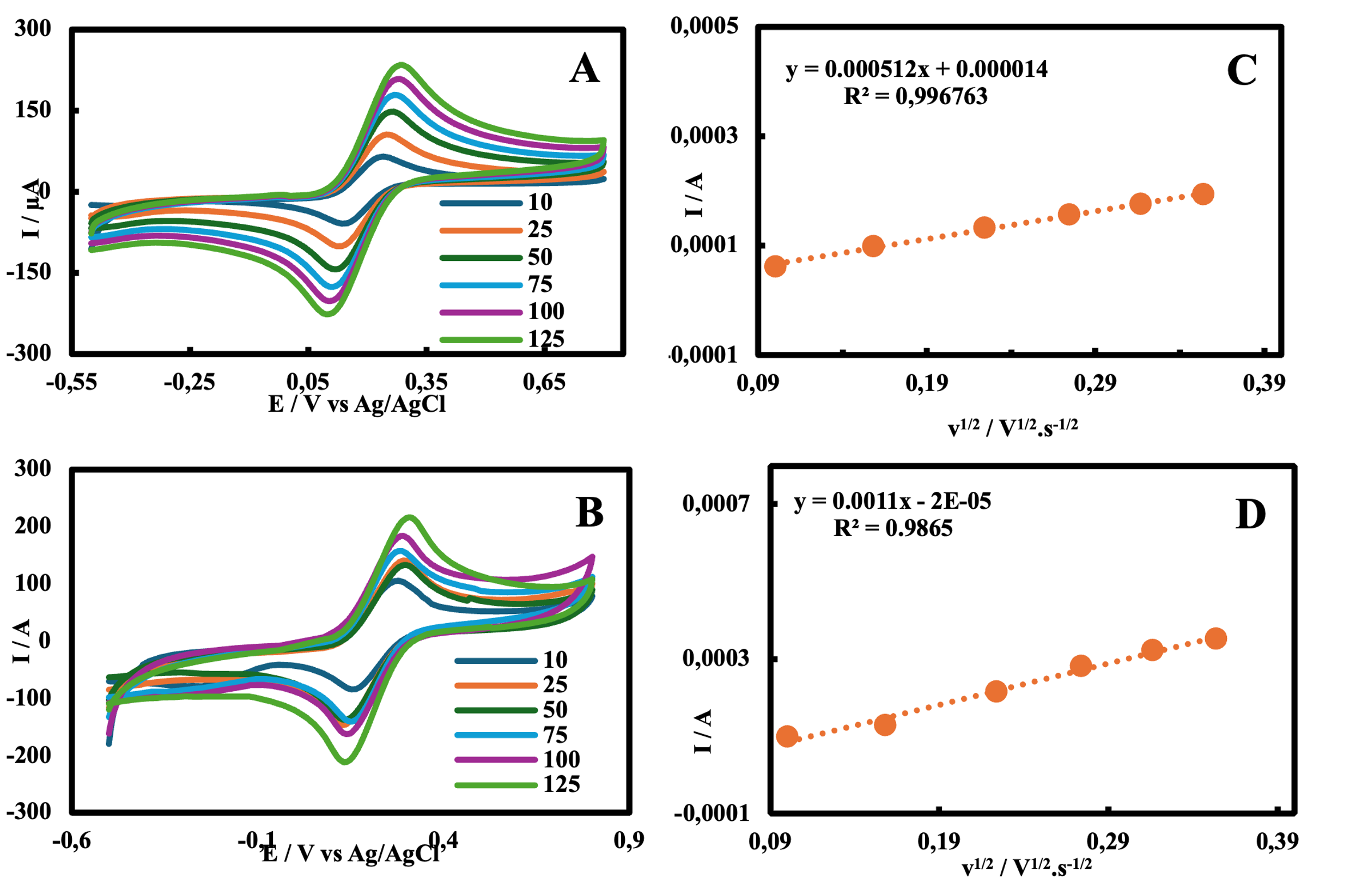


**Fig. S1** CVs of single SPE **(A)** and single HSPE **(B)** in redox probe solution at different scan rates (10, 25, 50, 75, 100, 125 mV s^-1^), graphs of peak current-square root of scan rate obtained from CVs **(C, D)**

**Table S1** Data obtained from CVs, DPVs, and EISs (only single) of single and quadruple HSPE and SPEs

| **Single HSPE** | | | | | | | **Aea (cm^2^)** |
| --- | --- | --- | --- | --- | --- | --- | --- |
| **HSPE** | **CV** | | **Ipa_avg_ (µA)** | | 145.74 (%RSD, %3.02) n=10 | | 0.2691 |
|  | **DPV** | | **Ipa_avg_ (µA)** | | 216.89 (%RSD, %4.33) n=10 | |  |
|  | **EIS** | | **Rct_avg_ (ohm)** | | 85.57 (%RSD, %3.34) n=10 | |  |
| **SPE** | **CV** | | **Ipa_avg_ (µA)** | | 133.76 (%RSD, %1.79) n=10 | | 0.1012 |
|  | **DPV** | | **Ipa_avg_ (µA)** | | 182.92 (%RSD, %2.40) n=10 | |  |
|  | **EIS** | | **Rct_avg_ (ohm)** | | 281.4 (%RSD, %1.64) n=10 | |  |
| **t_critical_** | **CV** | | 0.00042 | | | |  |
|  | **DPV** | | 0.0022 | | | |  |
|  | **EIS** | | 0.0006 | | | |  |
| **Quadruple HSPE** | | | | **CV** | | **DPV** | |
|  |  |  |  | **Ipa_ort_ (µA)** | | **Ipa_ort_ (µA)** | |
| **HSPE** | | **WE1** | | 84.01, (%RSD, %3.69) n=10 | | 101.50, (%RSD, %3.74) n=10 | |
|  |  | **WE2** | | 87.02, (%RSD, %4.73) n=10 | | 110.69, (%RSD, %3.01) n=10 | |
|  |  | **WE3** | | 84.35, (%RSD, %4.29) n=10 | | 115.16, (%RSD, %4.12) n=10 | |
|  |  | **WE4** | | 86.10, (%RSD, %4.30) n=10 | | 113.81, (%RSD, %3.12) n=10 | |
| **SPE** | | **WE1** | | 67.94, (%RSD, %3.14 n=10 | | 77.25, (%RSD, %4.87) n=10 | |
|  |  | **WE2** | | 69.05, (%RSD, %3.29) n=10 | | 71.60, (%RSD, %4.54) n=10 | |
|  |  | **WE3** | | 68.88, (%RSD, %3.90) n=10 | | 71.47, (%RSD, %4.74) n=10 | |
|  |  | **WE4** | | 68.21, (%RSD, %4.83) n=10 | | 70.99, (%RSD, %4.86) n=10 | |
| **t_critical_** | | **WE1** | | 3.77x10^-10^ | | | |
|  |  | **WE2** | | 1.7x10^-12^ | | | |
|  |  | **WE3** | | 7.4x10^-11^ | | | |
|  |  | **WE4** | | 7.2x10^-13^ | | | |

**2.2. AuNP modified electrodes**

To ensure sufficient AuNP formation to elicit significant electrochemical responses on HSPE surfaces, optimization studies were conducted for the solvent type of the HAuCl₄ solution, buffer pH, concentration, electrochemical method cycle count, and scan rate. To determine the optimal conditions, voltammograms and electrochemical impedance spectra were evaluated for electrochemical characterization, while SEM images and EDX analysis results were evaluated for morphological characterization. Electrochemical characterizations were conducted in a redox probe solution. The Aea of ​​the HSPEs was calculated using the Randles-Sevcik equation.

**For the optimization of the solvent type**, AuNPs were formed on the WE surface of HSPEs by the electrochemical method using HAuCl₄ solutions in pH 7.0 PBS (non-acidic) and 0.5 M H₂SO₄ pH 7.0 PBS (acidic). Table S2 shows the Ipa_avg_, Rct_avg_, ∆I_avg_, and ∆Rct_avg_ values ​​of HSPE and HSPE/AuNPs. ∆I_avg_ and ∆Rct_avg_ values ​​directly reveal the effect of AuNP modification. According to Table S2, the highest ∆Ipa_avg_ values ​​were obtained in the acid-free PBS solvent system. At the same time, according to the results obtained from the EIS, the highest ∆Rct_avg_ value was also obtained in the acid-free PBS solvent system. These results show that the HSPEs coated in a non-acidic medium exhibit significantly higher conductivity. The SEM images of the acidic and non-acidic AuNPs formed on the HSPEs surface, shown in Figures S2A-D, show that AuNPs formed homogeneously on the HSPE/AuNP _pH 7.0 PBS (non-acidic)_ surface. In contrast, it is clearly seen that AuNP formation was not observed in the HSPE/AuNP _H₂SO₄ pH 7.0 PBS (acidic)_. Furthermore, the EDX analyses in Figures S2 C and D support the SEM images, showing that Au is only present in the HSPE/AuNP _pH 7.0 PBS (non-acidic)_. These results indicate that the acid-free solvent system is more suitable for the successful and homogeneous deposition of AuNPs on the HSPEs surface.

**Table S2** Data obtained from the CVs, DPVs, and EISs of single HSPEs and HSPE/AuNP electrodes prepared using different solvent systems

|  | **CV** | | **DPV** | | **EIS** | |
| --- | --- | --- | --- | --- | --- | --- |
| **Formulation** | **Ipa_avg_**  **(µA)** | **Ipa_avg_**  **(µA)** | **Ipa_avg_**  **(µA)** | **Ipa_avg_**  **(µA)** | **Rct_avg_**  **(ohm)** | **Rct_avg_**  **(ohm)** |
| **HSPE** | 145.8 | 145.8 | 216.9 | 216.9 | 85.57 | 85.57 |
|  | **pH 7.0 H_2_SO_4_ (acidic)** | **pH 7.0 PBS (non-acidic)** | **pH 7.0 H_2_SO_4_ (acidic)** | **pH 7.0 PBS (non-acidic)** | **pH 7.0 H_2_SO_4_ (acidic)** | **pH 7.0 PBS (non-acidic)** |
| **HSPE/AuNP** | 214.7 | 248.8 | 243.2 | 281.8 | 61.6 | 15.6 |
|  | **∆I_avg_ (µA)** | | | | **∆Rct_avg_ (ohm)** | |
|  | 68.9 | 103.1 | 26.3 | 64.9 | 23.97 | 69.97 |


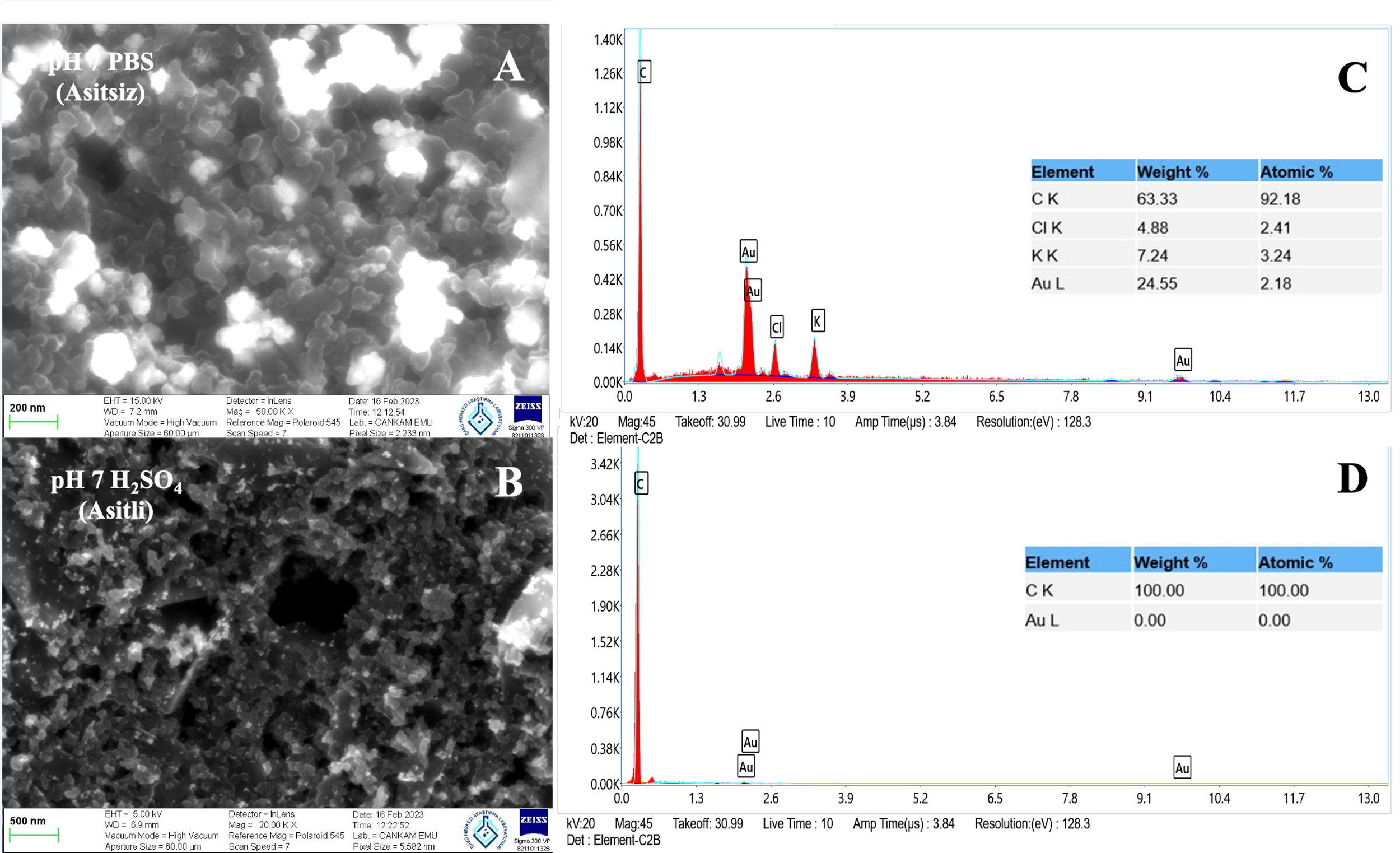


**Fig. S2** SEM images of HSPE/AuNP _H₂SO₄ pH 7.0 PBS_ _(acidic)_ **(A)** and HSPE/AuNP _pH_ _7.0 PBS (non-acidic)_ **(B)**, EDX analyses of HSPE/AuNP _H₂SO₄ pH 7.0 PBS_ _(acidic)_ **(C)** and HSPE/AuNP _pH_ _7.0 PBS (non-acidic)_ **(D)**

**To optimize the buffer solution pH**, HAuCl₄ solutions were prepared at 50 mM concentrations in three different PBS solutions at pH 6, 7, and 8, and AuNPs were formed on the surface of HSPEs (Table S3). According to the CV, DPV, and EIS data in Table S3, Ipa_avg_ values ​​increased with AuNP modification in all pH buffer systems, while Rct_avg_ values ​​decreased and conductivity increased. The highest ∆I_avg_ and ∆Rct_avg_ values ​​were obtained with the HSPE/AuNP _pH 7.0 PBS_ electrode prepared using a pH 7.0 PBS solution. These results suggest that the pH 7.0 PBS solution is the optimum environment for the reduction of HAuCl₄ on the HSPEs surface and the formation of AuNPs. When the SEM images of AuNP-modified electrodes prepared at different pHs are examined (Figure S3A-C), it is seen that they are in excellent agreement with the electrochemical results. It is clearly seen that the HSPE/AuNPs produced using HAuCl₄ prepared in a pH 7.0 PBS solution have a more homogeneous and uniform distribution of AuNPs on the PBS surface. This homogeneous distribution allows for the exposure of more active centers and, thus, more efficient electrochemical reactions. In contrast, at pH 8, it is clearly seen that the AuNPs form agglomerates on the electrode surface. The agglomeration of nanoparticles reduces the total active surface area and may hinder electron transfer. The results of EDX analysis (Figure 3D-F), performed to verify the elemental distribution of homogeneous and bulk structures, prove that both homogeneous and bulk structures on the surfaces are composed of the Au element. These results indicate that the observed structures are AuNPs and that pH affects not only the formation of the particles but also their morphological arrangement on the surface [2].

**Table S3** Data obtained from the CVs, DPVs, and EISs of single HSPE and HSPE/AuNP electrodes prepared using HAuCl₄ in PBS at different pH

|  | **CV** | | | **DPV** | | | **EIS** | | |
| --- | --- | --- | --- | --- | --- | --- | --- | --- | --- |
| **Formulation** | **Ipa_avg_**  **(µA)** | **Ipa_avg_**  **(µA)** | **Ipa_avg_**  **(µA)** | **Ipa_avg_**  **(µA)** | **Ipa_avg_**  **(µA)** | **Ipa_avg_**  **(µA)** | **Rct_avg_**  **(ohm)** | **Rct_avg_**  **(ohm)** | **Rct_avg_**  **(ohm)** |
| **HSPE** | 145.8 | 145.8 | 145.8 | 216.9 | 216.9 | 216.9 | 85.57 | 85.57 | 85.57 |
|  | **pH 6.0** | **pH 7.0** | **pH 8.0** | **pH 6.0** | **pH 7.0** | **pH 8.0** | **pH 6.0** | **pH 7.0** | **pH 8.0** |
| **HSPE/AuNP** | 205.3 | 248.8 | 215.1 | 228.4 | 281.8 | 250.2 | 38.2 | 15.6 | 29.5 |
|  | **∆I_avg_ (µA)** | | | | | | **∆Rct_avg_ (ohm)** | | |
| **∆I** | 59.55 | 103.05 | 69.35 | 11.45 | 64.9 | 33.3 | 47.37 | 69.97 | 56.07 |


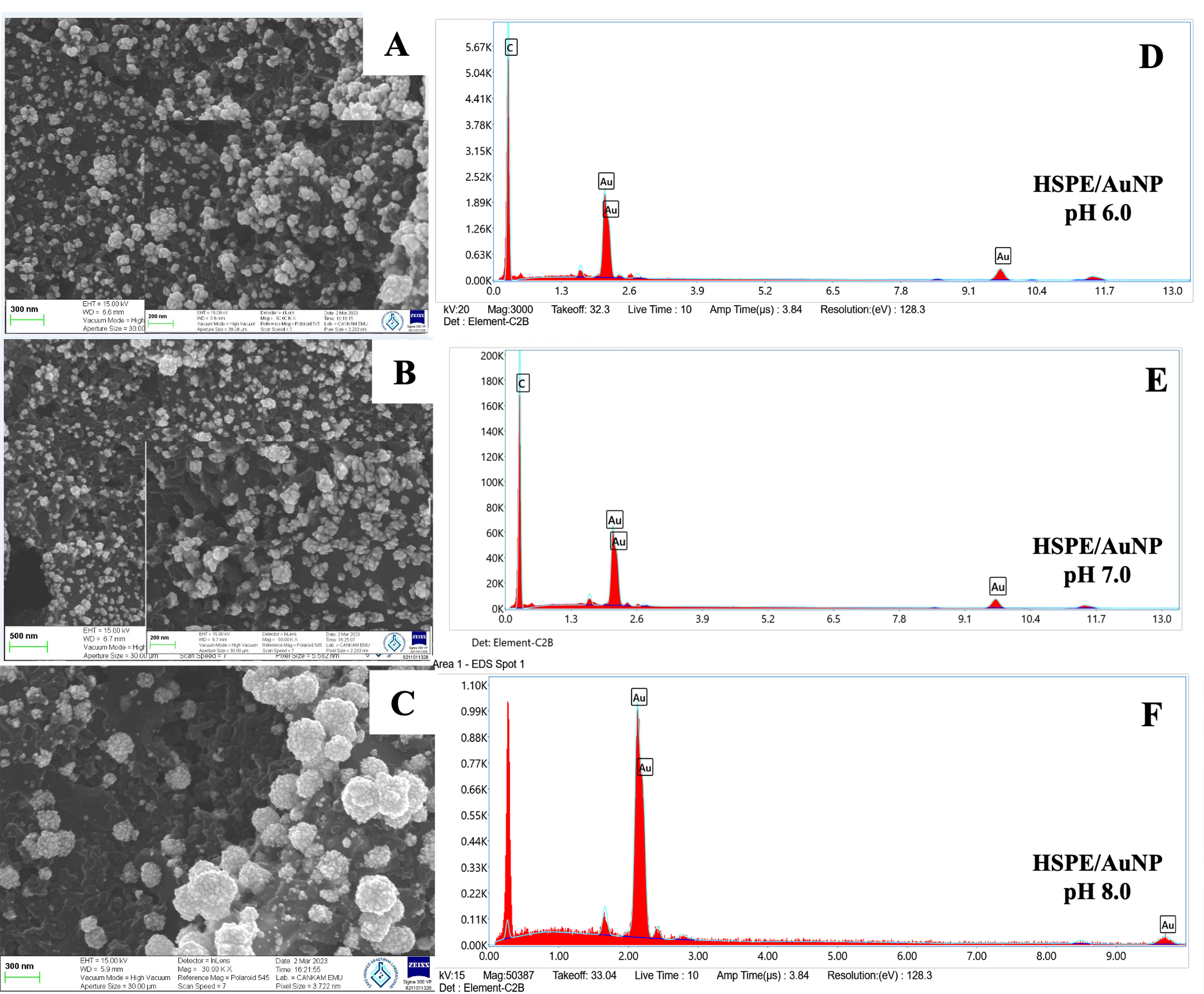


**Fig. S3** SEM images of HSPE/AuNP _pH 6.0_ **(A)**, HSPE/AuNP _pH 7.0_ **(B),** HSPE/AuNP _pH 8.0_ (**C),** and EDX analysis results of HSPE/AuNP _pH 6.0_ **(D),** HSPE/AuNP _pH 7.0_ **(E),** HSPE/AuNP _pH 8.0_ **(F)**

**To optimize the cycle number of the CV method**, AuNP-modified electrodes were prepared at four different cycle numbers by applying 5, 10, 15, and 20 CV cycles, and AuNPs were formed on the surface of the HSPEs. According to the CV, DPV, and EIS data in Table S4, it is observed that there is a significant increase in Ip_avg_ values ​​and a significant decrease in Rct_avg_ values ​​with the AuNP modification at all cycle numbers. Among the different cycle numbers examined, the highest ∆I_avg_ and ∆Rct_avg_ values ​​were obtained at the HSPE/AuNP (10n) electrode, indicating that the 10n AuNP modification provides the most suitable conditions. The Aea of ​​the HSPE and AuNP-modified HSPEs was calculated using the Randles-Sevcik equation and the plots of the root cause of the scan rate versus peak currents given in Figure S4F-J [1]. The Aea of ​​the HSPEs was calculated as 0.2691 cm². The Aea of ​​the HSPE/AuNP electrodes was found to be 0.4403 cm² (5 n), 0.8317 cm² (10 n), 0.5626 cm² (15 n), and 0.2446 cm² (20 n), respectively, and it was understood that the electrode with the highest Aea was the HSPE/AuNP (10 n) electrode. High Aea increases current responses by providing more points (active sites) on the electrode where redox reactions can occur. However, the decrease in surface area at 15 and 20 cycles suggests that excessive AuNP accumulation leads to agglomeration of the particles, thus reducing the active surface [3]. When the SEM images of AuNP-modified HSPEs prepared at different cycle numbers are examined (Figure S5A-D), a homogeneous distribution is observed, and the images are similar. In the SEM images of HSPE/AuNPs prepared at 15 and 20 cycles, it is clearly seen that the AuNPs accumulate on the surface in bulk. According to the EDX analysis results (Figure S5E-H), it is clearly seen that the nanoparticles formed on the electrode surface consist of the Au element. Based on the electrochemical characterization and SEM-EDX analysis results, 10 n was selected as the optimum cycle number in the CV method applied for the modification of AuNPs.

**Table S4** Data obtained from the CVs, DPVs, and EISs of single HSPE and HSPE/AuNPs prepared with different cycle numbers (5n, 10n, 15n, and 20n)

|  | **CV** | | | | **DPV** | | | | **EIS** | | | |
| --- | --- | --- | --- | --- | --- | --- | --- | --- | --- | --- | --- | --- |
| **Formulation** | **Ipa_avg_**  **(µA)** | **Ipa_avg_**  **(µA)** | **Ipa_avg_**  **(µA)** | **Ipa_avg_**  **(µA)** | **Ipa_avg_**  **(µA)** | **Ipa_avg_**  **(µA)** | **Ipa_avg_**  **(µA)** | **Ipa_avg_**  **(µA)** | **Rct_avg_**  **(ohm)** | **Rct_avg_**  **(ohm)** | **Rct_avg_**  **(ohm)** | **Rct_avg_**  **(ohm)** |
| **HSPE** | 145.8 | 145.8 | 145.8 | 145.8 | 216.9 | 216.9 | 216.9 | 216.9 | 85.57 | 85.57 | 85.57 | 85.57 |
|  | **5n** | **10n** | **15n** | **20n** | **5n** | **10n** | **15n** | **20n** | **5n** | **10n** | **15n** | **20n** |
| **HSPE/AuNP** | 242.62 | 248.8 | 227.77 | 223.33 | 250.55 | 281.8 | 263.2 | 242.2 | 72.6 | 15.6 | 40.8 | 65.7 |
|  | **∆I_avg_ (µA)** | | | | | | | | **∆Rct_avg_ (ohm)** | | | |
|  | 96.87 | 103.05 | 82.02 | 77.58 | 33.65 | 64.9 | 46.3 | 25.3 | 12.97 | 69.97 | 64.1 | 19.87 |
| **HSPE**  **Aea (cm^2^)** | 0.2691 | | | |  | | |  |  | | | |
| **HSPE/AuNP**  **Aea (cm^2^)** | 0.4403 | 0.8317 | 0.5626 | 0.2446 |  |  |  |  |  | | | |

**
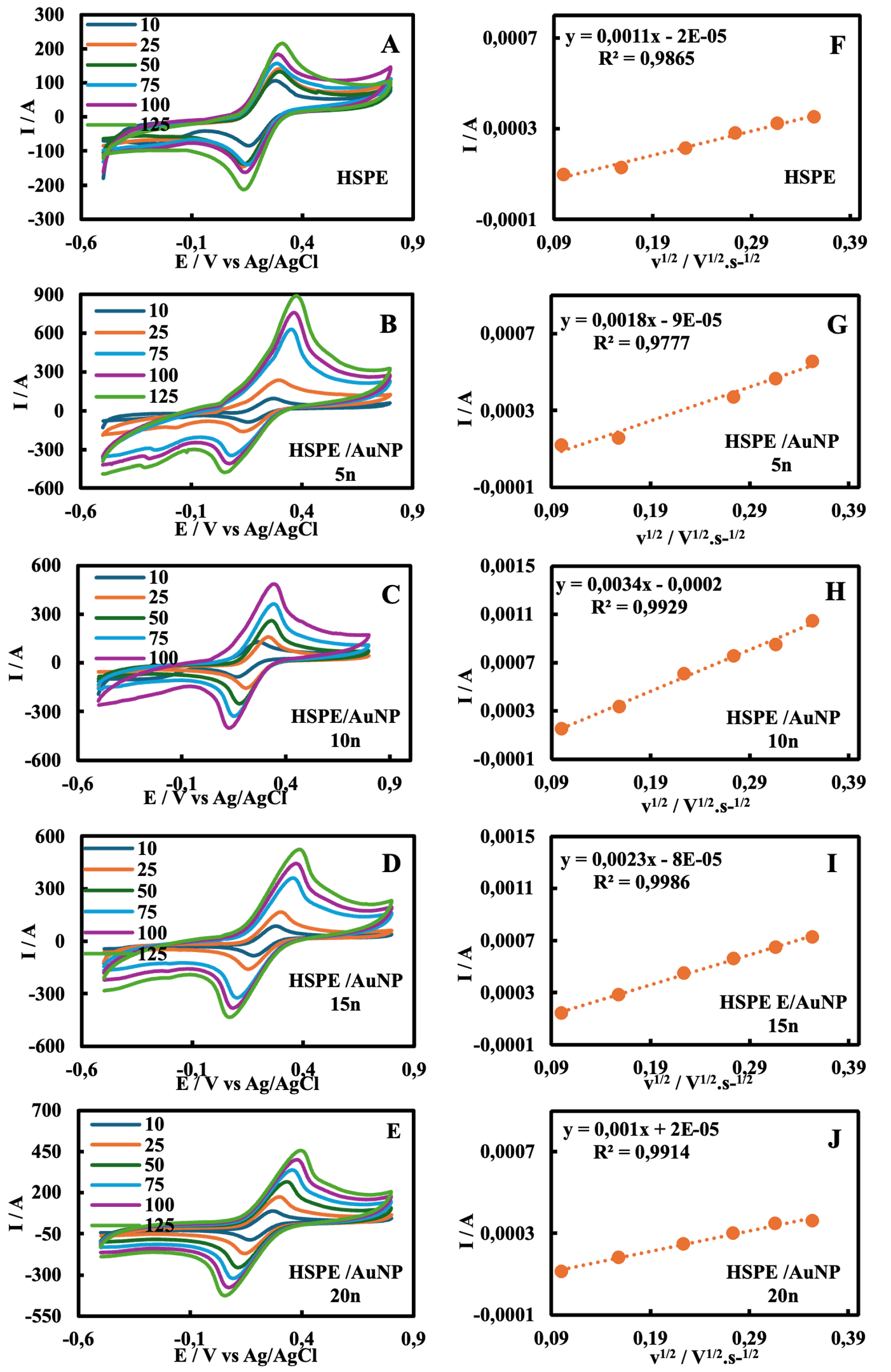
**

**Fig. S4** CVs of HSPE **(A),** HSPE/AuNP_5n_ **(B),** HSPE/AuNP_10n_ **(C),** HSPE/AuNP_15n_ **(D),** HSPE/AuNP_20n_ **(E)** in redox probe solution at different scan rates (10, 25, 50, 75, 100, 125 mV s^-1^), graphs of peak current-square root of scan rate obtained from CVs **(F-J)**

**
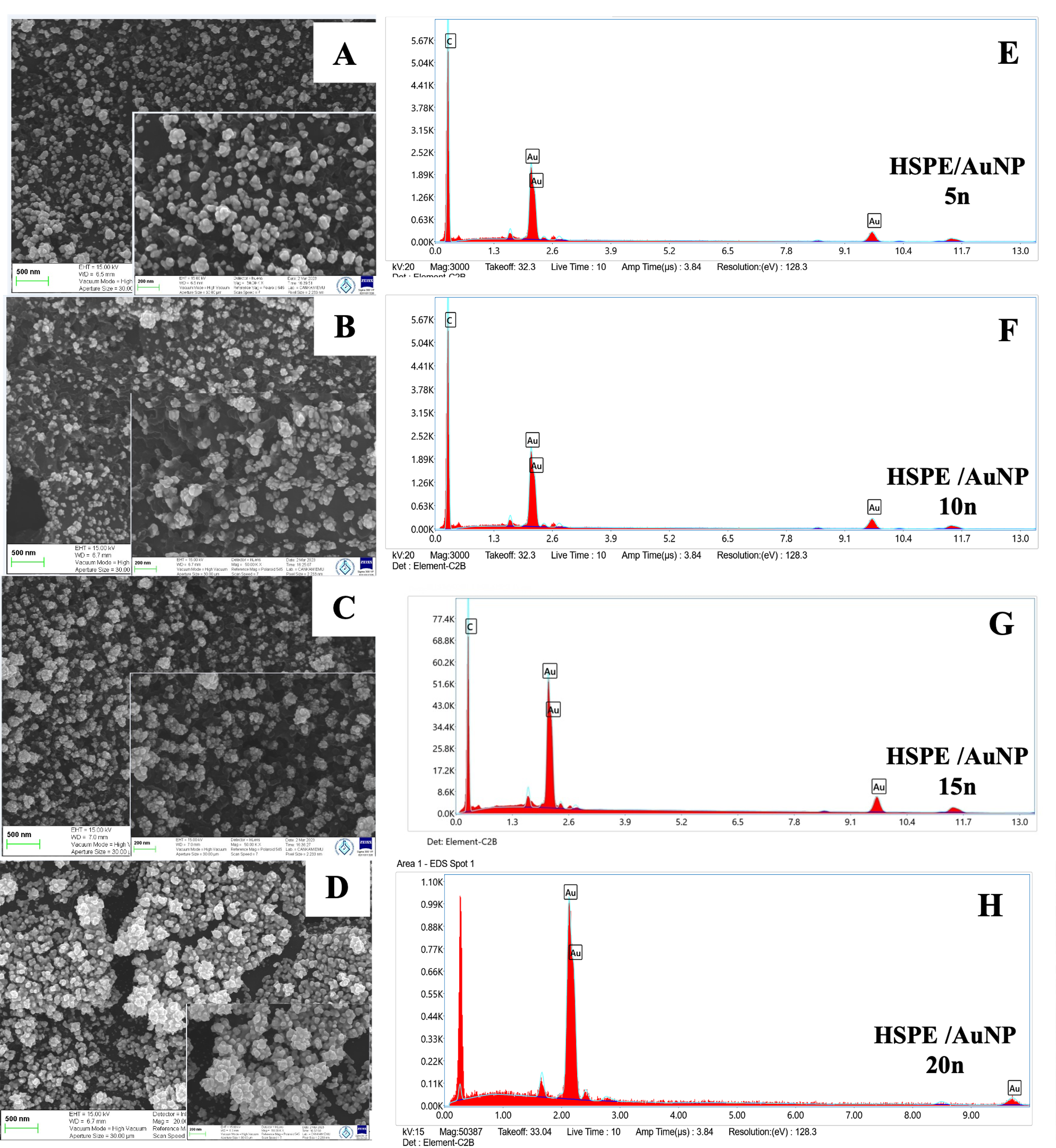
**

**Fig. S5** SEM images and EDX analysis of AuNP-modified HSPEs at different cycle numbers: 5n **(A-E)**, 10n **(B-F)**, 15n **(C-G)**, 20n **(D-H)**

**To optimize the scan rate of the CV method**, AuNP-modified HSPEs were prepared at three different scan rates by applying 10 cycles of CV at scan rates of 25, 50, and 100 mV s-1, and AuNPs were formed on the surface of the HSPEs. According to the CV, DPV, and EIS data in Table S5, it is observed that there is a significant increase in Ipa_avg_ values ​​and a significant decrease in Rct_avg_ values ​​with AuNP modification at all scan rates. The highest ∆I_avg_ and ∆Rct_avg_ values ​​were obtained in HSPE/AuNPs prepared at a scan rate of 50 mV s^-1^, indicating that a scan rate of 50 mV s^-1^ provides the most suitable conditions for AuNP modification. The CVs given in Figures S6A-D and S6E-H are plotted against the square root of the scan rate, and Aea's were calculated using the Randless-Sevcik equation. The Aea of ​​HSPE/AuNPs were found to be 0.5381 cm^2^ (25 mV s^-1^), 0.8072 cm^2^ (50 mV s^-1^), and 0.3118 cm^2^ (100 mV s^-1^). It was found that the electrode with the highest Aea value was in the HSPE prepared with AuNP modification at a scan rate of 50 mV s^-1^. When the SEM images of AuNP-modified HSPEs prepared at different scanning speeds were examined (Figure S7A-C), it was observed that the best nanoparticle distribution belonged to the electrodes prepared at a scanning speed of 50 mV s⁻¹. SEM images of the electrodes prepared at scan speeds of 25 mV s⁻¹ and 100 mV s⁻¹ show that AuNPs formed in stacked forms on the electrode surface. According to the EDX analysis results, it is clearly seen that the nanoparticles formed on the electrode surface are composed of the Au element (Figure S7D-F). According to the electrochemical characterization and SEM-EDX analysis results, the optimum scanning speed for the CV method applied for the modification of AuNPs was determined as 50 mV s⁻¹.

**Table S5** Data obtained from the CVs, DPVs, and EISs of single HSPE and HSPE/AuNP prepared at different scan rates (25, 50, and 100 mV s⁻¹)

|  | **CV** | | | **DPV** | | | **EIS** | | |
| --- | --- | --- | --- | --- | --- | --- | --- | --- | --- |
|  | **Ipa_avg_**  **(µA)** | **Ipa_avg_**  **(µA)** | **Ipa_avg_**  **(µA)** | **Ipa_avg_**  **(µA)** | **Ipa_avg_**  **(µA)** | **Ipa_avg_**  **(µA)** | **Rct_avg_**  **(ohm)** | **Rct_avg_**  **(ohm)** | **Rct_avg_**  **(ohm)** |
| **HSPE** | 145.8 | 145.8 | 145.8 | 216.9 | 216.9 | 216.9 | 85.57 | 85.57 | 85.57 |
|  | **25**  **mV s^-1^** | **50**  **mV s^-1^** | **100**  **mV s^-1^** | **25**  **mV s^-1^** | **50**  **mV s^-1^** | **100**  **mV s^-1^** | **25**  **mV s^-1^** | **50**  **mV s^-1^** | **100**  **mV s^-1^** |
| **HSPE/AuNP** | 240.1 | 248.8 | 224.68 | 251.61 | 281.9 | 240.1 | 29.8 | 15.6 | 39.4 |
|  | **∆I_avg_ (µA)** | | | | | | **∆Rct_avg_ (ohm)** | | |
|  | 94.35 | 103.05 | 78.93 | 34.71 | 64.9 | 14.59 | 55.77 | 69.97 | 46.17 |
| **HSPE**  **Aea (cm^2^)** | 0.2691 | | |  |  |  |  | | |
| **HSPE/AuNP**  **Aea (cm^2^)** | 0.5382 | 0.8317 | 0.3180 |  |  |  |  | | |


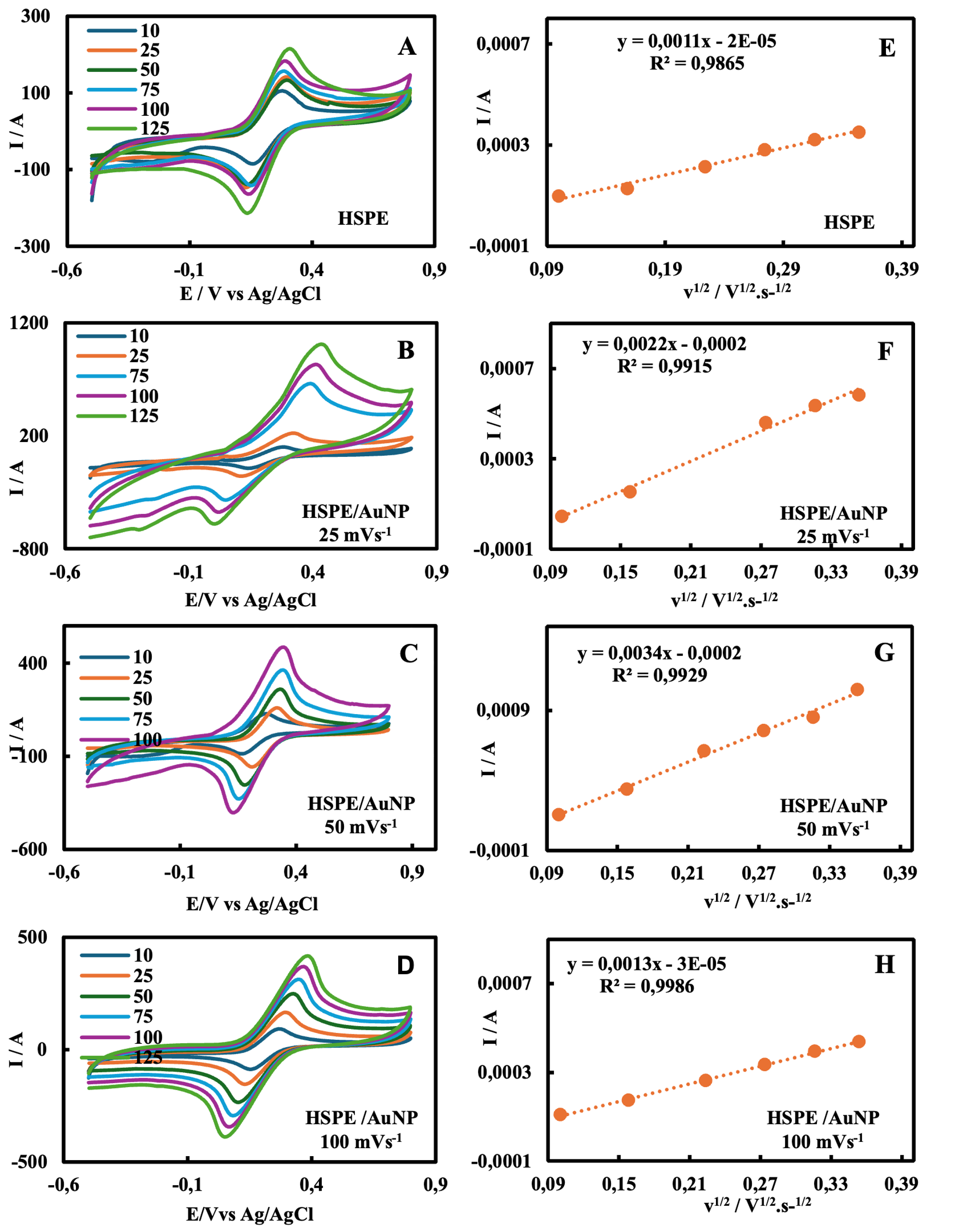


**Fig. S6** CVs of HSPE **(A),** HSPE/AuNP (25 mV s^-1^) **(B),** HSPE/AuNP (50 mV s^-1^) **(C),** HSPE/AuNP (100 mV s^-1^) **(D)** in redox probe solution at different scan rates (10, 25, 50, 75, 100, 125 mV s^-1^), graphs of peak current-square root of scan rate obtained from CVs **(E-H)**

**
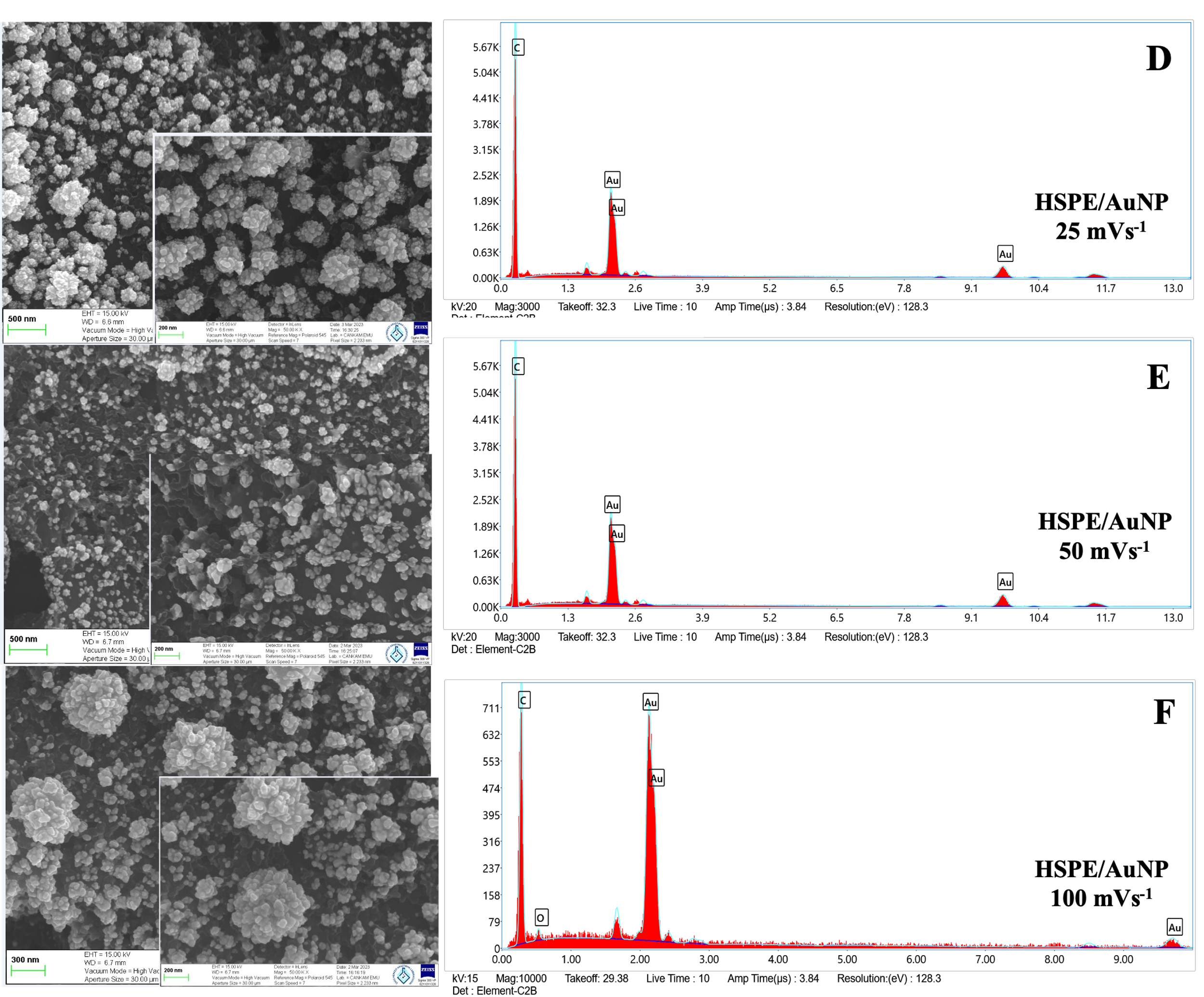
**

**Fig. S7** SEM images and EDX analysis of AuNP-modified HSPEs prepared at scan rates of 25 mV s^-1^ **(A-D)**, 50 mV s^-1^ **(B-E)**, 100 mV s^-1^ **(C-F)**

**To optimize the concentration of the HAuCl₄ solution**, solutions with different HAuCl₄ concentrations (0.6 mM, 4 mM, 6 mM, 60 mM, and 600 mM) were prepared in pH 7.0 PBS, and AuNPs were formed on the surface of HSPEs. According to the CV, DPV, and EIS data in Table S6, at all HAuCl₄ concentrations, Ipa_avg_ values ​​increased with AuNP modification, while Rct_avg_ values ​​decreased, and the conductivity of the electrodes increased. According to the CV and DPV data, the highest ∆I_avg_ was obtained with the 600 mM HAuCl₄ concentration, and according to the EIS data, the highest ∆Rct_avg_ value was obtained with the electrodes prepared with the 4 mM HAuCl₄ concentration. CVs are given in Figures S8A-F, the plots against the square root of the scan rate are given in Figures S8G-L, and Aea's were calculated using the Randless-Sevcik equation. The Aea of ​​HSPE/AuNPs were found to be 0.5137 cm² (0.6 mM), 0.8317 cm² (4 mM), 0.2935 cm² (6 mM), 0.5870 cm² (60 mM), and 1.4922 cm² (600 mM). It was understood that the HSPE/AuNP electrode with the highest surface area was the electrode modified with 600 mM HAuCl₄ concentration. When the SEM images of AuNP-modified HSPEs prepared in different HAuCl₄ solution concentrations are examined, it is seen that the AuNP distribution on the surface of HSPE/AuNPs prepared with 0.6 mM HAuCl₄ solution is sparse compared to HSPE/AuNPs prepared with 4 mM HAuCl₄ solution (Figure S9A-B). In the SEM images of HSPE/AuNPs prepared with 6 mM and 60 mM HAuCl₄ solutions, it is clearly seen that AuNPs are present in bulk on the electrode surface (Figure S9C-D). On the surface of HSPE/AuNPs prepared with 600 mM HAuCl₄ solution, it is clearly seen that AuNPs move away from the nanoparticle size and form microstructures, and the structure on the electrode surface is also disrupted (Figure S9E). According to the EDX analysis results, it is clearly seen that the nanoparticles and macrostructures formed on the electrode surface are composed of the Au element (Figure S9F-J). According to the electrochemical characterization results, although the highest Aea and ∆Iavg values ​​were obtained with HSPE/AuNPs prepared with 600 mM HAuCl₄ concentration, the optimum HAuCl₄ concentration was selected as 4 mM to prepare AuNP-modified HSPEs, considering the microstructures in HSPE/AuNPs prepared with 600 mM HAuCl₄ concentration in SEM images.

**Table S6** Data obtained from the CVs, DPVs, and EISs of single HSPE and HSPE/AuNP prepared with different HAuCl₄ concentrations

|  | **CV** | | | | | **DPV** | | | | | | **EIS** | | | | |
| --- | --- | --- | --- | --- | --- | --- | --- | --- | --- | --- | --- | --- | --- | --- | --- | --- |
|  | **Ipa_avg_**  **(µA)** | **Ipa_avg_**  **(µA)** | **Ipa_avg_**  **(µA)** | **Ipa_avg_**  **(µA)** | **Ipa_avg_**  **(µA)** | **Ipa_avg_**  **(µA)** | **Ipa_avg_**  **(µA)** | **Ipa_avg_**  **(µA)** | | **Ipa_avg_**  **(µA)** | **Ipa_avg_**  **(µA)** | **Rct_avg_**  **(ohm)** | **Rct_avg_**  **(ohm)** | **Rct_avg_**  **(ohm)** | **Rct_avg_**  **(ohm)** | **Rct_avg_**  **(ohm)** |
| **HSPE** | 145.8 | 145.8 | 145.8 | 145.8 | 145.8 | 216.9 | 216.9 | 216.9 | | 216.9 | 216.9 | 85.57 | 85.57 | 85.57 | 85.57 | 85.57 |
|  | **0.6**  **mM** | **4**  **mM** | **6**  **mM** | **60**  **mM** | **600 mM** | **0.6**  **mM** | **4**  **mM** | **6**  **mM** | | **60**  **mM** | **600 mM** | **0.6**  **mM** | **4**  **mM** | **6**  **mM** | **60**  **mM** | **600 mM** |
| **HSPE/AuNP** | 204.7 | 248.8 | 226.4 | 236.9 | 977.1 | 233.6 | 281.9 | 204.7 | | 248.8 | 226.4 | 79.1 | 15.6 | 63.5 | 40.6 | 39.5 |
|  | **∆I_avg_ (µA)** | | | | | | | | **∆Rct_avg_ (ohm)** | | | | | | | |
|  | 58.95 | 103.1 | 80.65 | 91.15 | 831.4 | 16.7 | 64.9 | 29.1 | | 40.05 | 161 | 6.47 | 69.97 | 22.07 | 44.97 | 46.07 |
| **HSPE**  **Aea (cm^2^)** | 0.2691 | | | | |  |  |  | |  |  |  |  |  |  |  |
| **HSPE/AuNP**  **Aea (cm^2^)** | 0.513 | 0.831 | 0.293 | 0.587 | 1.492 |  |  |  | |  |  |  |  |  |  |  |


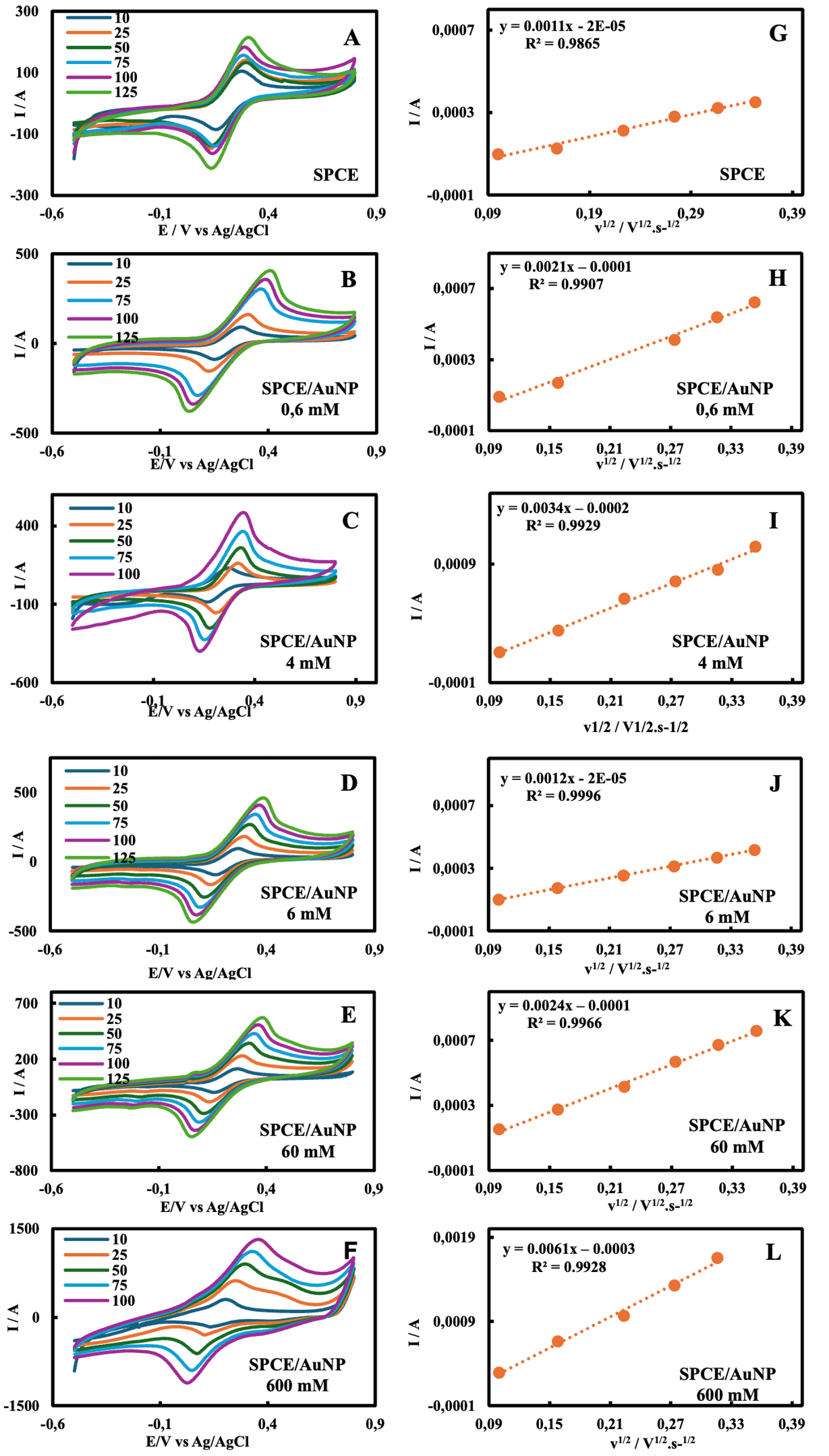


**Fig. S8** CVs of bare HSPE and HSPE/AuNPs prepared with different HAuCl_4_ concentrations **(A)**, 0.6 mM **(B)**, 4 mM **(C)**, 6 mM **(D)**, 60 mM **(E)**, 600 mM **(F)** in redox probe solution at different scan rates (10, 25, 50, 75, 100, 125 mV s^-1^), graphs of peak current-square root of scan rate obtained from CVs **(G-L)**


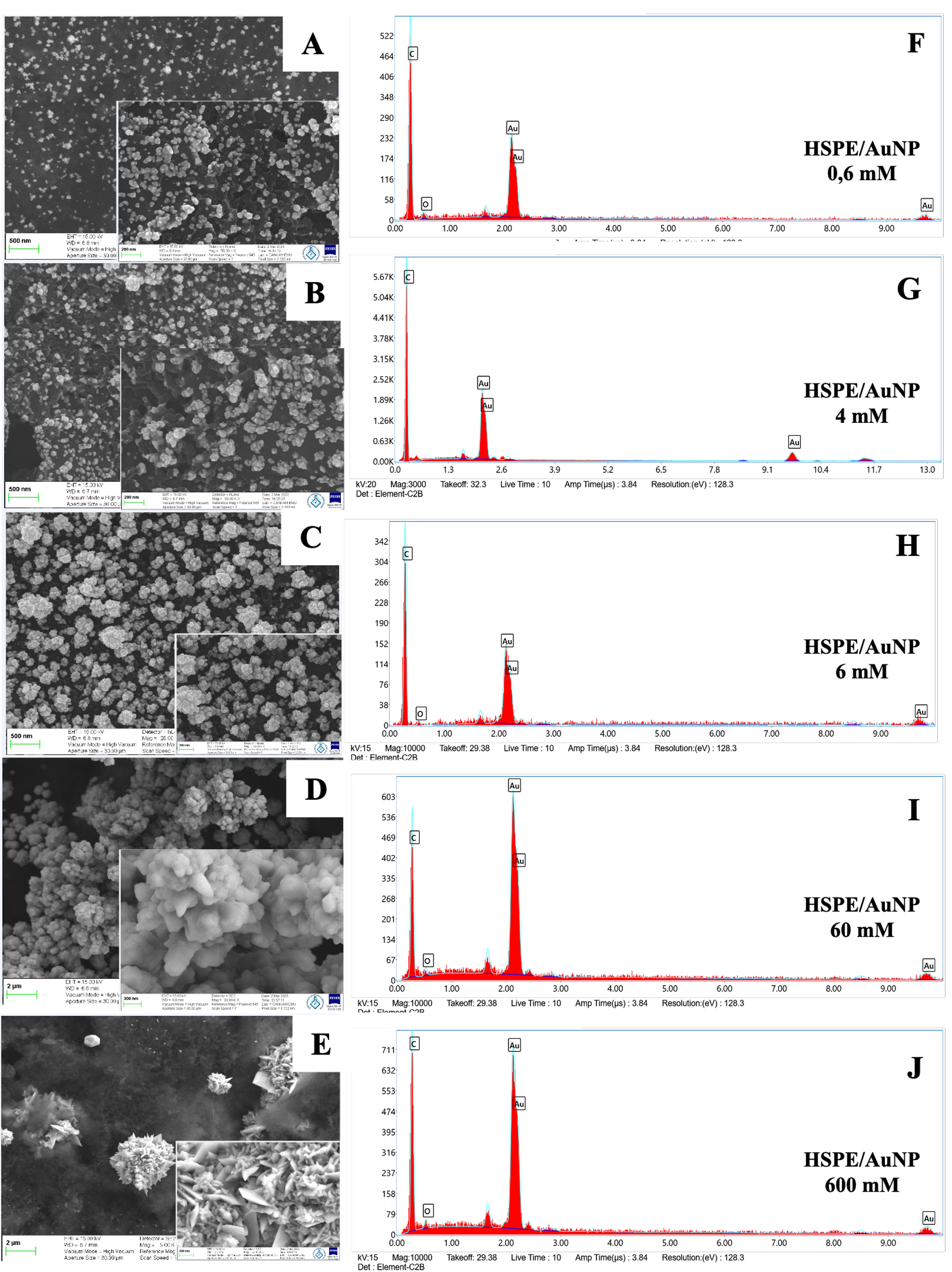


**Fig. S9** SEM images and EDX analysis of HSPE/AuNPs prepared with different HAuCl_4_ concentrations, 0.6 mM **(A-F)**, 4 mM **(B-G)**, 6 mM **(C-H)**, 60 mM **(D-I)**, 600 mM **(E-J)**

SAM films of 3-MPA and 6-MHA were formed on the HSPE/AuNP surface at **different incubation times (6 hours, 12 hours, and 18 hours), and optimization studies** were carried out (Table S7). According to the CV and DPV data obtained from Table S7, a decrease in the Ipa_avg_ values ​​of the electrodes on which SAM layers were formed with 3-MPA and 6-MHA at different incubation times was observed, while according to EIS data, an increase in Rct_avg_ values ​​was observed. As the incubation time increased (from 6 hours to 12 and 18 hours), a thicker SAM layer was formed, and diffusion decreased. The highest ∆I_avg_ and ∆Rct_avg_ values ​​were obtained for HSPE/AuNP prepared with an incubation time of 18 hours. Based on these results, the optimum incubation time for both 3-MPA and 6-MHA was determined as 18 hours.

**Table S7** Data obtained from CVs, DPVs, and EISs of single HSPE/AuNP incubated with 50 mM 3-MPA and 6-MHA for different times (6, 12, and 18 hours) during the preparation steps

|  |  | **CV** | | | **DPV** | | | **EIS** | | |
| --- | --- | --- | --- | --- | --- | --- | --- | --- | --- | --- |
|  | **Formulation** | **6 h** | **12 h** | **18 h** | **6 h** | **12 h** | **18 h** | **6 h** | **12 h** | **18 h** |
|  |  | **Ipa_avg_**  **(µA)** | **Ipa_avg_**  **(µA)** | **Ipa_avg_**  **(µA)** | **Ipa_avg_**  **(µA)** | **Ipa_avg_**  **(µA)** | **Ipa_avg_**  **(µA)** | **Rct_avg_**  **(ohm)** | **Rct_avg_**  **(ohm)** | **Rct_avg_**  **(ohm)** |
| **3-MPA** | **AuNP** | 248.8 | 248.8 | 248.8 | 281.9 | 281.9 | 281.9 | 85.57 | 85.57 | 85.57 |
|  | **3-MPA** | 225.1 | 220.5 | 218.9 | 286.2 | 273.0 | 250.6 | 36.5 | 27.97 | 21.8 |
|  |  | **∆I_avg_ (µA)** | | | | | | **∆Rct_avg_ (ohm)** | | |
|  |  | 23.7 | 28.3 | 29.9 | 4.3 | 8.9 | 31.3 | 49.07 | 57.6 | 63.77 |
| **6-MHA** | **AuNP** | 248.8 | 248.8 | 248.8 | 281.9 | 281.9 | 281.9 | 85.57 | 85.57 | 85.57 |
|  | **6-MHA** | 220.4 | 204.9 | 199.6 | 278.5 | 264.9 | 245.5 | 31.41 | 24.4 | 15.6 |
|  |  | **∆I_avg_ (µA)** | | | | | | **∆Rct_avg_ (ohm)** | | |
|  |  | 28.4 | 43.9 | 49.2 | 3.4 | 17 | 36.5 | 54.17 | 61.17 | 69.97 |
|  |  |  |  |  |  |  |  |  |  |  |

SAM films of 3-MPA and 6-MHA were formed on the HSPE/AuNP surface **at different concentrations (25 mM, 50 mM, and 100 mM), and optimization studies** were conducted (Table S8). According to the CV and DPV data obtained from Table S8, thicker SAM layers were formed as the concentrations of 3-MPA and 6-MHA increased. These results indicate that as the concentrations increased (50 mM and 100 mM), the SAM films created a partial barrier on the electrode surface, reducing diffusion. Data obtained from CV, DPV, and EIS indicate that a concentration of 25 mM for both sulfur compounds was insufficient to form SAM layers. This suggests that at low concentrations, a sufficient number of mercaptan molecules could not adsorb on the electrode surface, or the formed SAM layer was not sufficient and uniform enough to provide complete surface coverage. According to these results, since the optimum concentrations of 3-MPA and 6-MHA (50 mM and 100 mM) could not be definitely decided, a single AGR2 immunosensor was prepared up to the antibody step (Table S9). HSPE/AuNP/6-MHA and HPCE/AuNP/3-MHA were activated with EDC-NHS and then incubated with anti-AGR2. Electrochemical characterizations of the electrodes in the EDC-NHS and anti-AGR2 steps were carried out (by CV, DPV, and EIS methods). According to the CV, DPV, and EIS data given in Table S9, the highest ∆I_avg_ and ∆Rct_avg_ values ​​were obtained in the 6-MHA sulfur compound at a concentration of up to 100 mM. At a concentration of 100 mM 6-MHA, conductivity decreased due to the formation of denser SAM layers on the electrode surface, which limited diffusion. Based on these results, the optimum concentration was determined as 100 mM, and 6-MHA was the best-performing sulfur compound.

**Table S8** Data obtained from CVs, DPVs, and EISs of single HSPE/AuNP modified with 3-MPA and 6-MHA at different concentrations (25, 50, and 100 mM) during the preparation steps

|  | **Formulation** | **CV** | | | **DPV** | | | **EIS** | | |
| --- | --- | --- | --- | --- | --- | --- | --- | --- | --- | --- |
|  |  | **25 mM** | **50 mM** | **100 mM** | **25 mM** | **50 mM** | **100 mM** | **25 mM** | **50 mM** | **100 mM** |
|  |  | **Ipa_avg_**  **(µA)** | **Ipa_avg_**  **(µA)** | **Ipa_avg_**  **(µA)** | **Ipa_avg_**  **(µA)** | **Ipa_avg_**  **(µA)** | **Ipa_avg_**  **(µA)** | **Rct_avg_**  **(ohm)** | **Rct_avg_**  **(ohm)** | **Rct_avg_**  **(ohm)** |
| **3-MPA** | **AuNP** | 248.8 | 248.8 | 248.8 | 281.9 | 281.9 | 281.9 | 85.57 | 85.57 | 85.57 |
|  | **3MPA** | 224.5 | 218.9 | 201.4 | 261.2 | 250.6 | 238.1 | 29.7 | 54.1 | 26.37 |
|  |  | **∆I_avg_ (µA)** | | | | | | **∆Rct_avg_ (ohm)** | | |
|  |  | 24.3 | 29.9 | 47.4 | 20.7 | 31.3 | 43.8 | 49.07 | 31.47 | 63.77 |
| **6-MHA** | **AuNP** | 248.8 | 248.8 | 248.8 | 281.9 | 281.9 | 281.9 | 85.57 | 85.57 | 85.57 |
|  | **6MHA** | 195.6 | 199.6 | 192.8 | 247.3 | 245.5 | 224.2 | 15.6 | 41.5 | 50.7 |
|  |  | **∆I_avg_ (µA)** | | | | | | **∆Rct_avg_ (ohm)** | | |
|  |  | 53.2 | 49.2 | 56 | 64.9 | 36.5 | 51 | 69.97 | 44.07 | 34.87 |

**Table S9** Data obtained from CVs, DPVs, and EISs of EDC-NHS and anti-AGR2 electrodes prepared with HSPE/AuNP electrodes incubated with 3-MPA and 6-MHA at concentrations of 50 and 100 mM

|  |  | **CV** | | **DPV** | | **EIS** | |
| --- | --- | --- | --- | --- | --- | --- | --- |
|  |  | **50 mM** | **100 mM** | **50 mM** | **100 mM** | **50 mM** | **100 mM** |
|  |  | **Ipa_avg_**  **(µA)** | **Ipa_avg_**  **(µA)** | **Ipa_avg_**  **(µA)** | **Ipa_avg_**  **(µA)** | **Rct_avg_**  **(ohm)** | **Rct_avg_**  **(ohm)** |
| **3-MPA** | **EDC-NHS** | 97.4 | 167.4 | 34.4 | 108.8 | 280.9 | 260.5 |
|  | **Anti-AGR2** | 96.5 | 145.9 | 29.6 | 87.0 | 304.9 | 218.2 |
|  |  | **∆I_avg_ (µA)** | | | | **∆Rct_avg_ (ohm)** | |
|  |  | 0.84 | 21.5 | 4.8 | 21.8 | 24.0 | 42.3 |
| **6-MHA** | **EDC-NHS** | 66.4 | 291.4 | 57.9 | 113.9 | 253.0 | 239.5 |
|  | **Anti-AGR2** | 47.9 | 268.1 | 55.5 | 78.9 | 293.7 | 302.7 |
|  |  | **∆I_avg_ (µA)** | | | | **∆Rct_avg_ (ohm)** | |
|  |  | 18.5 | 23.3 | 2.4 | 35.0 | 40.7 | 63.2 |

SEM, FT-IR, and XPS analyses of HSPE/AuNP/6-MHA/EDC-NHS were performed. SEM images showed that the surface of the HSPE/AuNP/6-MHA/EDC-NHS modified electrode had a rough structure (Figure S10A), and EDX analysis showed the presence of the nitrogen element on the surface (Figure S10B) [4]. According to these results, the electrode surfaces were activated with EDC-NHS. The absorbance band observed at 1614.200 cm⁻¹ in the FT-IR spectrum originates from the COO-NHS ester group obtained by replacing the carbonyl group with the amide group (Figure S10C) [4]. The C1s, N1s, and O1s peaks obtained in the XPS analysis of the HSPE/AuNP/6-MHA/EDC-NHS electrode prepared under optimum conditions prove the presence of EDC-NHS on the surface (Figure S10D) [5].


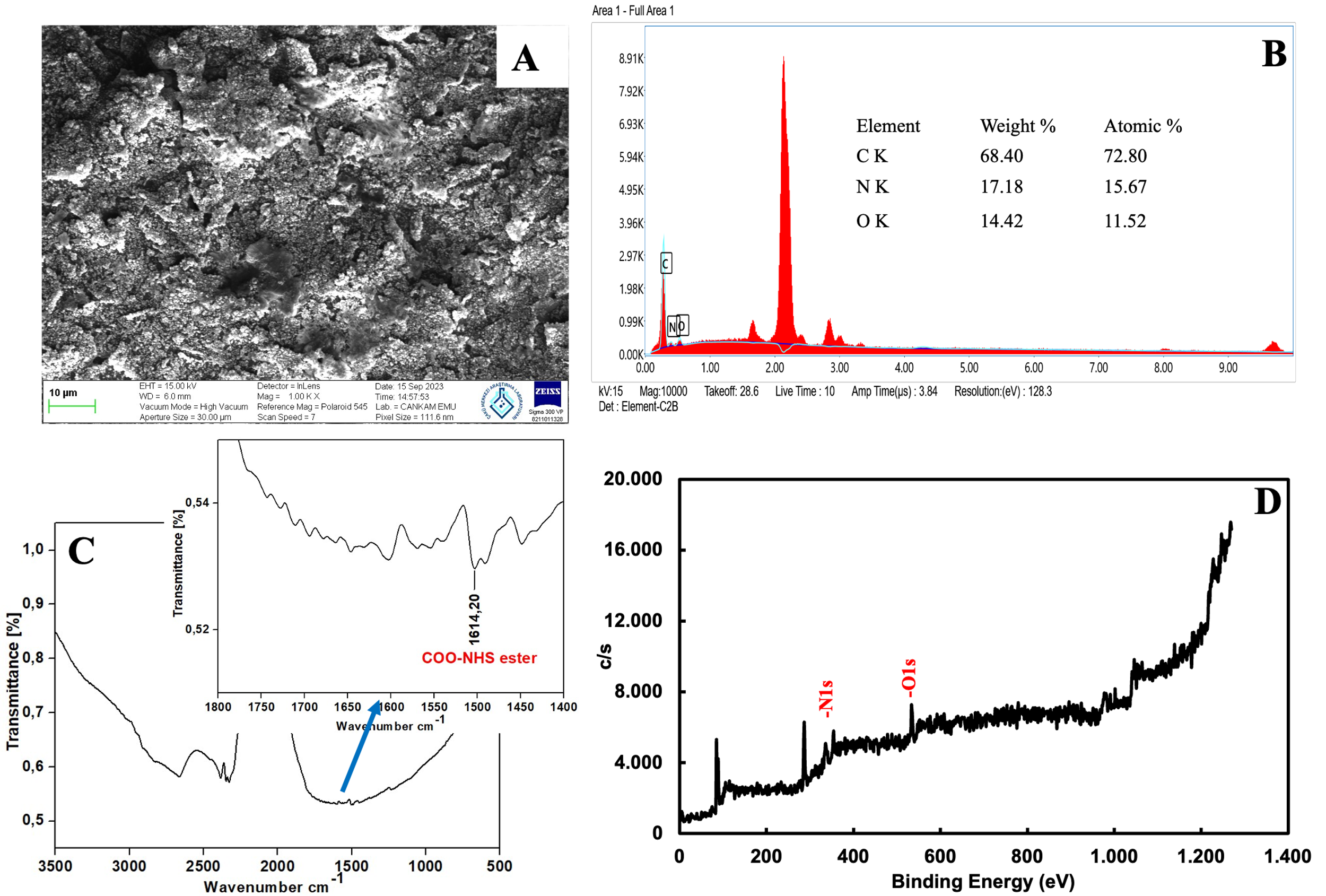


**Fig. S10** SEM-EDX **(A, B),** FT-IR **(C)**, and XPS **(D)** analysis of single HSPE/AuNP/6-MHA/EDC-NHSs

**Table S10** Epa_avg_, Ipa_avg,_ and Rct_avg_ values ​ at each preparation step of single and panel AGR2, GLY, FOLR1, and SMRP immunosensors

| **AGR2 immunosensors** | | | | | | | | | | | | | | | | |
| --- | --- | --- | --- | --- | --- | --- | --- | --- | --- | --- | --- | --- | --- | --- | --- | --- |
|  | **CV** | | | **DPV** | | | | | | | | **EIS** | | |  |  |
| **Formulation** | **Epa_avg_ (V)** | **Ipa_avg_ (µA)** | | | **Epa_avg_ (V)** | | | | | **Ipa_avg_ (µA)** | | | **Rct_avg_ (ohm)** | | |  |
| EDC-NHS | 0.314 | | 128.5 | | | | 0.185 | | | 86.5 | | | | 239.5 | |  |
| Anti-AGR2 | 0.416 | | 102.7 | | | | 0.180 | | | 68.5 | | | | 302.7 | |  |
| BSA | 0.434 | | 100.1 | | | | 0.185 | | | 56.8 | | | | 340.8 | |  |
| AGR2 | 0.434 | | 86.2 | | | | 0.190 | | | 39.6 | | | | 368.1 | |  |
| **GLY immunosensors** | | | | | | | | | | | | | | | | |
|  | **CV** | | | | | **DPV** | | | | | **EIS** | | | |  |  |
| **Formulation** | **Epa_avg_ (V)** | | **Ipa_avg_ (µA)** | | | | **Epa_avg_ (V)** | | | **Ipa_avg_ (µA)** | | | | **Rct_avg_ (ohm)** | |  |
| EDC-NHS | 0.382 | | 133.72 | | | | 0.235 | | | 63.48 | | | | 208.3 | |  |
| Anti-GLY | 0.394 | | 121.93 | | | | 0.230 | | | 48.88 | | | | 271.3 | |  |
| BSA | 0.412 | | 108.55 | | | | 0.230 | | | 40.61 | | | | 318.7 | |  |
| GLY | 0.446 | | 96.15 | | | | 0.240 | | | 27.30 | | | | 374.0 | |  |
| **FOLR1 immunosensors** | | | | | | | | | | | | | | | | |
|  | **CV** | | | | | | | **DPV** | | | | **EIS** | | |  |  |
| **Formulation** | **Epa_avg_ (V)** | **Ipa_avg_ (µA)** | | | | | | | **Epa_avg_ (V)** | **Ipa_avg_ (µA)** | | | **Rct_avg_ (ohm)** | | |  |
| EDC-NHS | 0.362 | 147.79 | | | | | | | 0.230 | 77.93 | | | 260.7 | | |  |
| Anti-FOLR1 | 0.376 | 130.89 | | | | | | | 0.230 | 63.49 | | | 295.5 | | |  |
| BSA | 0.364 | 121.34 | | | | | | | 0.230 | 61.42 | | | 301.4 | | |  |
| FOLR1 | 0.432 | 104.25 | | | | | | | 0.235 | 30.75 | | | 326.6 | | |  |
| **SMRP immunosensors** | | | | | | | | | | | | | | | | |
|  | **CV** | | | | | | | | **DPV** | | | | **EIS** | | |  |
| **Formulation** | **Epa_avg_ (V)** | **Ipa_avg_ (µA)** | | | | | | | **Epa_avg_ (V)** | **Ipa_avg_ (µA)** | | | **Rct_avg_ (ohm)** | | |  |
| EDC-NHS | 0.374 | 139.29 | | | | | | | 0.220 | 76.91 | | | 351.2 | | |  |
| Anti-SMRP | 0.376 | 123.50 | | | | | | | 0.225 | 54.78 | | | 376.8 | | |  |
| BSA | 0.398 | 114.92 | | | | | | | 0.225 | 48.51 | | | 728.9 | | |  |
| SMRP | 0.396 | 103.61 | | | | | | | 0.235 | 36.12 | | | 751.6 | | |  |
| **Panel immunosensors** | | | | | | | | | | | | | | | | |
|  | **Formulation** | **CV** | | | | | | | | **DPV** | | | | | |  |
|  |  | **Epa_ort_ (V)** | | | | | | | **Ipa_ort_ (µA)** | **Epa_ort_ (V)** | | | **Ipa_ort_ (µA)** | | |  |
| **WE1**  **AGR2 immunosensors** | EDC-NHS | 0.274 | | | | | | | 119.99 | 0.222 | | | 64.42 | | |  |
|  | Anti-AGR2 | 0.302 | | | | | | | 103.69 | 0.230 | | | 39.69 | | |  |
|  | BSA | 0.304 | | | | | | | 101.55 | 0.220 | | | 44.09 | | |  |
|  | AGR2 | 0.314 | | | | | | | 84.59 | 0.215 | | | 31.91 | | |  |
|  | **Formulation** | **CV** | | | | | | | | **DPV** | | | | | |  |
|  |  | **Epa_ort_ (V)** | | | | | | | **Ipa_ort_ (µA)** | **Epa_ort_ (V)** | | | **Ipa_ort_ (µA)** | | |  |
| **WE2**  **GLY immunosensors** | EDC-NHS | 0.298 | | | | | | | 112.49 | 0.235 | | | 59.35 | | |  |
|  | Anti-AGR2 | 0.312 | | | | | | | 97.26 | 0.245 | | | 46.39 | | |  |
|  | BSA | 0.326 | | | | | | | 93.65 | 0.235 | | | 57.57 | | |  |
|  | AGR2 | 0.326 | | | | | | | 74.45 | 0.240 | | | 39.63 | | |  |
|  | **Formulation** | **CV** | | | | | | | | **DPV** | | | | | |  |
|  |  | **Epa_ort_ (V)** | | | | | | | **Ipa_ort_ (µA)** | **Epa_ort_ (V)** | | | **Ipa_ort_ (µA)** | | |  |
| **WE3**  **SMRP immunosensors** | **EDC-NHS** | 0.306 | | | | | | | 109.47 | 0.235 | | | 73.92 | | |  |
|  | **Anti-SMRP** | 0.312 | | | | | | | 91.50 | 0.240 | | | 46.50 | | |  |
|  | **BSA** | 0.324 | | | | | | | 88.17 | 0.240 | | | 58.95 | | |  |
|  | **SMRP** | 0.320 | | | | | | | 69.28 | 0.245 | | | 45.46 | | |  |
|  | **Formulation** | **CV** | | | | | | | | **DPV** | | | | | |  |
|  |  | **Epa_ort_ (V)** | | | | | | | **Ipa_ort_ (µA)** | **Epa_ort_ (V)** | | | **Ipa_ort_ (µA)** | | |  |
| **WE4**  **FOLR1 immunosensors** | **EDC-NHS** | 0.312 | | | | | | | 109.24 | 0.240 | | | 54.42 | | |  |
|  | **Anti-FOLR1** | 0.330 | | | | | | | 89.40 | 0.235 | | | 30.15 | | |  |
|  | **BSA** | 0.334 | | | | | | | 88.88 | 0.235 | | | 40.15 | | |  |
|  | **FOLR1** | 0.330 | | | | | | | 66.57 | 0.245 | | | 26.23 | | |  |

**Table S11** Analytical performance data of single and panel AGR2, GLY, FOLR1, and SMRP immunosensors

| **Single immunosensors** | | | |
| --- | --- | --- | --- |
| **Analyte** | **Linear range** | **Sensitivity** | **LOD** |
| AGR2 | 1- 400 pg mL^-1^ | 0.0307 µA mL pg^-1^ | 0.309 pg mL^-1^ |
| GLY | 1- 500 pg mL^-1^ | 0.0625 µA mL pg^-1^ | 0.360 pg mL^-1^ |
| FOLR1 | 1- 400 pg mL^-1^ | 0.0618 µA mL pg^-1^ | 0.307 pg mL^-1^ |
| SMRP | 1- 500 pg mL^-1^ | 0.0767 µA mL pg^-1^ | 0.334 pg mL^-1^ |
| **Panel immunosensor** | | | |
| **Analyte** | **Linear range** | **Sensitivity** | **LOD** |
| AGR2 | 0.1- 500 pg mL^-1^ | 0.0606 µA mL pg^-1^ | 0.343 pg mL^-1^ |
| GLY | 0.1- 500 pg mL^-1^ | 0.0753 µA mL pg^-1^ | 0.359 pg mL^-1^ |
| SMRP | 0.1- 500 pg mL^-1^ | 0.0800 µA mL pg^-1^ | 0.361 pg mL^-1^ |
| FOLR1 | 0.1- 500 pg mL^-1^ | 0.0792 µA mL pg^-1^ | 0.368 pg mL^-1^ |

**Table S12** Repeatability and reproducibility of single and panel AGR2, GLY, FOLR1, and SMRP immunosensors

| **Repeatability test results of single immunosensors** | | |
| --- | --- | --- |
| **Analyte** | | **RSD** |
| AGR2 | | % 4.22 (n=7), 50 ng mL^-1^ |
| GLY | | % 3.74 (n=7), 50 ng mL^-1^ |
| FOLR1 | | % 2.74 (n=7), 50 ng mL^-1^ |
| SMRP | | % 4.53 (n=7), 50 ng mL^-1^ |
| **Reproducibility test results of single immunosensors** | | |
| **Analyte** | | **RSD** |
| AGR2 | | % 4.36 (n=6), 50 ng mL^-1^ |
| GLY | | % 3.05 (n=6), 50 ng mL^-1^ |
| FOLR1 | | % 4.14 (n=6), 50 ng mL^-1^ |
| SMRP | | % 3.95 (n=6), 50 ng mL^-1^ |
| **Repeatability test results of panel immunosensors** | | |
| **Analyte** | | **RSD** |
| AGR2 | | %4.42 (n=6), 50 ng mL^-1^ |
| GLY | | %4.52 (n=6), 50 ng mL^-1^ |
| SMRP | | %4.78 (n=6), 50 ng mL^-1^ |
| FOLR1 | | %3.70 (n=6), 50 ng mL^-1^ |
| **Reproducibility test results of panel immunosensors** | | |
| **Analyte** | **RSD** | |
| AGR2 | %4.85 (n=6), 50 ng mL^-1^ | |
| GLY | %4.71 (n=6), 50 ng mL^-1^ | |
| SMRP | %3.74 (n=6), 50 ng mL^-1^ | |
| FOLR1 | %4.33 (n=6), 50 ng mL^-1^ | |

**Table S13** Results of analysis of AGR2, GLY, SMRP and FOLR1 antigens in commercial human serum samples by single and panel AGR2, GLY, FOLR1, and SMRP immunosensors

| **Single immunosensors** | | | | | | |
| --- | --- | --- | --- | --- | --- | --- |
| **Analyte** | **Antigen cons. (pg mL^-1^)** | **Added cons. (pg mL^-1^)** | **Calculated cons. (pg mL^-1^)** | **RSD (%)** | **% Recovery** | **Error (%)** |
| **AGR2** | 13.33 | 100 pg mL^-1^ | 110.40-113.96-119.19 | 4.35 | 101.05 | 1.05 |
|  |  | 200 pg mL^-1^ | 218.54-220.17-218.52 | 0.35 | 103.36 | 3.36 |
|  |  | 300 pg mL^-1^ | 315.34-326.03-322.45 | 1.30 | 103.90 | 3.30 |
| **GLY** | 89.14 | 100 pg mL^-1^ | 194.42-194.74-199.38 | 1.15 | 103.72 | 3.72 |
|  |  | 200 pg mL^-1^ | 284.34-292.50-284.34 | 1.34 | 99.28 | 0.71 |
|  |  | 300 pg mL^-1^ | 387.86-373.62-381.46 | 1.53 | 97.90 | 2.09 |
| **SMRP** | 8.54 | 100 pg mL^-1^ | 104.63-112.33-112.96 | 3.27 | 101.17 | 1.17 |
|  |  | 200 pg mL^-1^ | 213.24-211.41-218.13 | 1.32 | 102.86 | 2.86 |
|  |  | 300 pg mL^-1^ | 295.12-321.19-328.36 | 4.53 | 102.12 | 2.12 |
| **FOLR1** | 38.29 | 100 pg mL^-1^ | 143.41-144.22-140.19 | 1.22 | 103.51 | 3.51 |
|  |  | 200 pg mL^-1^ | 236.62-230.63-224.16 | 2.21 | 99.94 | 3.05 |
|  |  | 300 pg mL^-1^ | 335-363.16-339.85 | 3.55 | 102.7 | 4.35 |
| **Panel immunosensors** | | | | | | |
| **Analyte** | **Antigen cons.**  **(pg mL^-1^)** | **Added cons.**  **(pg mL^-1^)** | **Calculated cons.**  **(pg mL^-1^)** | **RSD (%)** | **% Recovery** | **Error (%)** |
| **WE1 (AGR2)** | 6.65 | 100 pg mL^-1^ | 143.41-144.22-140.19 | 1.22 | 103.51 | 3.51 |
|  |  | 200 pg mL^-1^ | 236.62-230.63-224.16 | 2.21 | 99.94 | 3.05 |
|  |  | 300 pg mL^-1^ | 335-363.16-339.85 | 355 | 102.7 | 4.35 |
| **WE2 (GLY)** | 92.36 | 100 pg mL^-1^ | 202.59-185.33-208.67 | 4.98 | 103.38 | 3.38 |
|  |  | 200 pg mL^-1^ | 297.94-286.92-265.94 | 4.68 | 97.00 | 3.00 |
|  |  | 300 pg mL^-1^ | 394.61-373.90-422.51 | 5.01 | 101.18 | 1.18 |
| **WE3 (SMRP)** | 9.93 | 100 pg mL^-1^ | 143.41-144.22-140.19 | 4.81 | 101.29 | 1.29 |
|  |  | 200 pg mL^-1^ | 236.62-230.63-224.16 | 3.03 | 99.92 | 0.08 |
|  |  | 300 pg mL^-1^ | 335-363.16-339.85 | 4.61 | 101.56 | 1.56 |
| **WE4 (FOLR1)** | 46.93 | 100 pg mL^-1^ | 153.25-150.72-149.84 | 0.95 | 102.95 | 2.95 |
|  |  | 200 pg mL^-1^ | 271.93-246.68-248.95 | 4.45 | 103.61 | 3.61 |
|  |  | 300 pg mL^-1^ | 338.22-336.07-317.76 | 2.78 | 95.32 | 4.68 |

**Table S14** Comparison of AGR2, GLY, SMRP, and FOLR1 analysis results performed with ELISA and single and panel immunosensors using t- and F-tests

| **Added AGR2 cons.** | **Single**  **immunosensor**  **(ng mL^-1^)** | **Panel**  **immunosensor**  **(ng mL^-1^)** | **ELISA**  **(ng mL^-1^)** | **F_critical_** | **F** | **F** | **t_critical_** | **t** | **p** | **t** | **p** |
| --- | --- | --- | --- | --- | --- | --- | --- | --- | --- | --- | --- |
|  |  |  |  |  | **Single immunosensor** | **Panel**  **immunosensor** |  | **Single immunosensor** | | **Panel immunosensor** | |
| 10 ng mL^-1^ | 9.71 | 9.80 | 10.12 | 19.5 | 0.37 | 15.01 | 4.3 | 3.11 | 0.089 | 0.45 | 0.69 |
|  | 10.06 | 9.93 | 9.65 |  |  |  |  |  |  |  |  |
|  | 10.59 | 9.91 | 10.12 |  |  |  |  |  |  |  |  |
| 20 ng mL^-1^ | 20.52 | 20.74 | 20.15 | 19.5 | 7.02 | 0.09 | 4.3 | 4.21 | 0.051 | 1.23 | 0.342 |
|  | 20.68 | 21.02 | 19.80 |  |  |  |  |  |  |  |  |
|  | 20.51 | 19.41 | 19.65 |  |  |  |  |  |  |  |  |
| 30 ng mL^-1^ | 30.26 | 29.84 | 29.84 | 19.5 | 0.82 | 0.97 | 4.3 | 3.02 | 0.094 | 2.41 | 0.136 |
|  | 31.27 | 30.57 | 30.56 |  |  |  |  |  |  |  |  |
|  | 30.91 | 30.71 | 30.71 |  |  |  |  |  |  |  |  |
| **Added GLY cons.** | **Single**  **immunosensor**  **(ng mL^-1^)** | **Panel**  **immunosensor**  **(ng mL^-1^)** | **ELISA**  **(ng mL^-1^)** | **F_critical_** | **F** | **F** | **t_critical_** | **t** | **p** | **t** | **p** |
|  |  |  |  |  | **Single immunosensor** | **Panel**  **immunosensor** |  | **Single immunosensor** | | **Panel immunosensor** | |
| 10 ng mL^-1^ | 10.53 | 11.02 | 10.59 | 19.5 | 2.78 | 0.14 | 4.3 | 1.3 | 0.323 | 0.48 | 0.677 |
|  | 10.56 | 9.30 | 9.92 |  |  |  |  |  |  |  |  |
|  | 11.02 | 11.62 | 10.81 |  |  |  |  |  |  |  |  |
| 20 ng mL^-1^ | 19.52 | 20.56 | 20.33 | 19.5 | 3.75 | 0.31 | 4.3 | 0.42 | 0.714 | 1.07 | 4.302 |
|  | 20.34 | 19.46 | 19.81 |  |  |  |  |  |  |  |  |
|  | 19.52 | 17.36 | 18.56 |  |  |  |  |  |  |  |  |
| 30 ng mL^-1^ | 29.87 | 30.23 | 30.36 | 19.5 | 0.21 | 0.02 | 4.3 | 3.19 | 0.086 | 0.34 | 0.765 |
|  | 28.45 | 28.15 | 29.75 |  |  |  |  |  |  |  |  |
|  | 29.23 | 33.02 | 29.86 |  |  |  |  |  |  |  |  |
| **Added FOLR1 cons.** | **Single**  **immunosensor**  **(ng mL^-1^)** | **Panel**  **immunosensor**  **(ng mL^-1^)** | **ELISA**  **(ng mL^-1^)** | **F_critical_** | **F** | **F** | **t_critical_** | **t** | **p** | **t** | **p** |
|  |  |  |  |  | **Single immunosensor** | **Panel**  **immunosensor** |  | **Single immunosensor** | | **Panel immunosensor** | |
| 100 pg mL^-1^ | 105.12 | 106.32 | 101.94 | 19.5 | 0.17 | 0.24 | 4.3 | 0.96 | 0.437 | 1.11 | 0.382 |
|  | 105.93 | 103.79 | 102.48 |  |  |  |  |  |  |  |  |
|  | 101.90 | 102.91 | 103.65 |  |  |  |  |  |  |  |  |
| 200 pg mL^-1^ | 198.33 | 225.00 | 201.45 | 19.5 | 0.05 | 0.01 | 4.3 | 2.76 | 0.109 | 1.23 | 0.344 |
|  | 192.34 | 199.75 | 199.65 |  |  |  |  |  |  |  |  |
|  | 185.87 | 202.02 | 198.65 |  |  |  |  |  |  |  |  |
| 300 pg mL^-1^ | 296.71 | 289.14 | 298.74 | 19.5 | 0.1 | 0.03 | 4.3 | 1.83 | 0.209 | 0.46 | 0.692 |
|  | 301.56 | 313.01 | 301.65 |  |  |  |  |  |  |  |  |
|  | 310.63 | 291.29 | 303.20 |  |  |  |  |  |  |  |  |
| **Added SMRP cons.** | **Single**  **immunosensor**  **(ng mL^-1^)** | **Panel**  **immunosensor**  **(ng mL^-1^)** | **ELISA**  **(ng mL^-1^)** | **F_critical_** | **F** | **F** | **t_critical_** | **t** | **p** | **t** | **p** |
|  |  |  |  |  | **Single immunosensor** | **Panel**  **immunosensor** |  | **Single immunosensor** | | **Panel immunosensor** | |
| 100 pg mL^-1^ | 96.09 | 96.5 | 98.73 | 19.5 | 1.71 | 0.85 | 4.3 | 0.26 | 0.813 | 0.14 | 0.902 |
|  | 103.79 | 108.87 | 98.74 |  |  |  |  |  |  |  |  |
|  | 104.42 | 98.87 | 109.24 |  |  |  |  |  |  |  |  |
| 200 pg mL^-1^ | 204.7 | 208.74 | 193.27 | 19.5 | 9.14 | 1.82 | 4.3 | 0.4 | 0.728 | 0.33 | 0.773 |
|  | 202.87 | 198.86 | 203.78 |  |  |  |  |  |  |  |  |
|  | 209.59 | 193.36 | 214.28 |  |  |  |  |  |  |  |  |
| 300 pg mL^-1^ | 286.58 | 289.61 | 308.82 | 19.5 | 0.36 | 3.05 | 4.3 | 0.19 | 0.864 | 1.58 | 0.255 |
|  | 312.65 | 295.36 | 319.32 |  |  |  |  |  |  |  |  |
|  | 319.82 | 301.64 | 298.31 |  |  |  |  |  |  |  |  |

**Table S15** Comparison of AGR2, GLY, SMRP, and FOLR1 analysis results performed with ELISA and single and panel immunosensors according to % difference values

| **Added AGR2**  **Cons.** | **Single**  **immunosensor**  **(ng mL^-1^)** | **Panel**  **immunosensor**  **(ng mL^-1^)** | **ELİSA**  **(ng mL^-1^)** | **Difference** | | **% Difference** | |
| --- | --- | --- | --- | --- | --- | --- | --- |
|  |  |  |  | **Single**  **immunosensor**  **(ng mL^-1^)** | **Panel**  **immunosensor**  **(ng mL^-1^)** | **Single**  **immunosensor** | **Panel**  **immunosensor** |
| 10 ng mL^-1^ | 10.12 | 9.88 | 9.96 | 0.154 | 0.084 | 1.52 | 0.85 |
| 20 ng mL^-1^ | 20.57 | 20.39 | 19.87 | 0.706 | 0.525 | 3.43 | 2.57 |
| 30 ng mL^-1^ | 30.81 | 29.34 | 30.38 | 0.439 | 1.034 | 1.42 | 3.52 |
| **Added GLY**  **Cons.** | **Single**  **immunosensor**  **(ng mL^-1^)** | **Panel**  **immunosensor**  **(ng mL^-1^)** | **ELİSA**  **(ng mL^-1^)** | **Difference** | | **% Difference** | |
|  |  |  |  | **Single**  **immunosensor**  **(ng mL^-1^)** | **Panel**  **immunosensor**  **(ng mL^-1^)** | **Individual**  **DPV** | **Panel**  **DPV** |
| 10 ng mL^-1^ | 10.70 | 10.64 | 10.44 | 0.26 | 0.21 | 2.5 | 1.9 |
| 20 ng mL^-1^ | 19.79 | 19.12 | 19.56 | 0.22 | 0.44 | 1.1 | 2.3 |
| 30 ng mL^-1^ | 29.18 | 30.46 | 29.98 | 0.80 | 0.48 | 2.7 | 1.6 |
| **Added FOLR1**  **Cons.** | **Single**  **immunosensor**  **(ng mL^-1^)** | **Panel**  **immunosensor**  **(ng mL^-1^)** | **ELİSA**  **(ng mL^-1^)** | **Difference** | | **% Difference** | |
|  |  |  |  | **Single**  **immunosensor**  **(ng mL^-1^)** | **Panel**  **immunosensor**  **(ng mL^-1^)** | **Individual**  **DPV** | **Panel**  **DPV** |
| 100 pg mL^-1^ | 104.32 | 104.34 | 102.69 | 1.63 | 1.65 | 1.6 | 1.6 |
| 200 pg mL^-1^ | 192.18 | 208.92 | 199.92 | 7.74 | 9.0 | 4.0 | 4.3 |
| 300 pg mL^-1^ | 302.97 | 297.81 | 301.20 | 1.77 | 3.4 | 0.6 | 1.1 |
| **Added SMRP**  **Cons.** | **Single**  **immunosensor**  **(ng mL^-1^)** | **Panel**  **immunosensor**  **(ng mL^-1^)** | **ELİSA**  **(ng mL^-1^)** | **Difference** | | **% Difference** | |
|  |  |  |  | **Single**  **immunosensor**  **(ng mL^-1^)** | **Panel**  **immunosensor**  **(ng mL^-1^)** | **Individual**  **DPV** | **Panel**  **DPV** |
| 100 pg mL^-1^ | 101.43 | 101.41 | 102.24 | 0.80 | 0.82 | 0.8 | 0.8 |
| 200 pg mL^-1^ | 205.72 | 200.32 | 203.78 | 1.94 | 3.5 | 0.9 | 1.7 |
| 300 pg mL^-1^ | 306.35 | 295.54 | 308.82 | 2.47 | 13.3 | 0.8 | 4.5 |

**CRediT authorship contribution statement**

**Melike BİLGİ KAMAÇ:** Conceptualization, Methodology, Investigation, Validation, Formal analysis, Data curation, Supervision, Writing- Original Draft, Funding acquisition, Resources, Project administration. **Ayşenur YILMAZ KABACA:** Conceptualization, Methodology, Investigation, Formal analysis, Data curation, Validation, Visualization, Writing-Original Draft. **Merve YILMAZ ÇILÇAR:** Methodology, Investigation, Validation, Formal analysis, Visualization, Writing-Original Draft. **Mustafa Kemal SEZGİNTÜRK:** Conceptualization, Methodology, Writing – review & editing. **Muhammed ALTUN:** Methodology, Investigation, Formal analysis.

**Declaration of competing interest**

The authors declare that they have no known competing financial interests or personal relationships that could have appeared to influence the work reported in this paper.

**Acknowledgments**

This work was supported by the Scientific and Technological Research Institution of Turkey (TUBITAK) 1001 (project number: 122Z426). Ayşenur YILMAZ KABACA and Merve YILMAZ ÇILÇAR thank the financial support from the TUBITAK under the BIDEB/2211-A Ph.D. Scholarship Programs.

**References**

1. Bard AJ, Faulkner LR, White HS. (2022) Electrochemical methods: fundamentals and applications. John Wiley & Sons.
2. Li C, Li D, Wan G, et al. (2011) Facile synthesis of concentrated gold nanoparticles with low size-distribution in water: temperature and pH controls. Nanoscale Research Letters 6:440. doi:10.1186/1556-276X-6-440
3. Zakaria ND, Omar MH, Ahmad Kamal NN, et al. (2021) Effect of supporting background electrolytes on the nanostructure morphologies and electrochemical behaviors of electrodeposited gold nanoparticles on glassy carbon electrode surfaces. ACS Omega 6:24419–24431. <https://doi.org/10.1021/acsomega.1c02670>
4. Rath D, Kumar S, Panda S. (2019) pH-based detection of target analytes in diluted serum samples using surface plasmon resonance immunosensor. Applied Biochemistry and Biotechnology 187:1272–1284. 9) https://doi.org/10.1007/s12010-018-2883-3
5. Azioune A, Ben Slimane A, Ait Hamou L, et al. (2004) Synthesis and characterization of active ester-functionalized polypyrrole− silica nanoparticles: application to the covalent attachment of proteins. Langmuir 20:3350–3356. <https://doi.org/10.1021/la030407s>

**
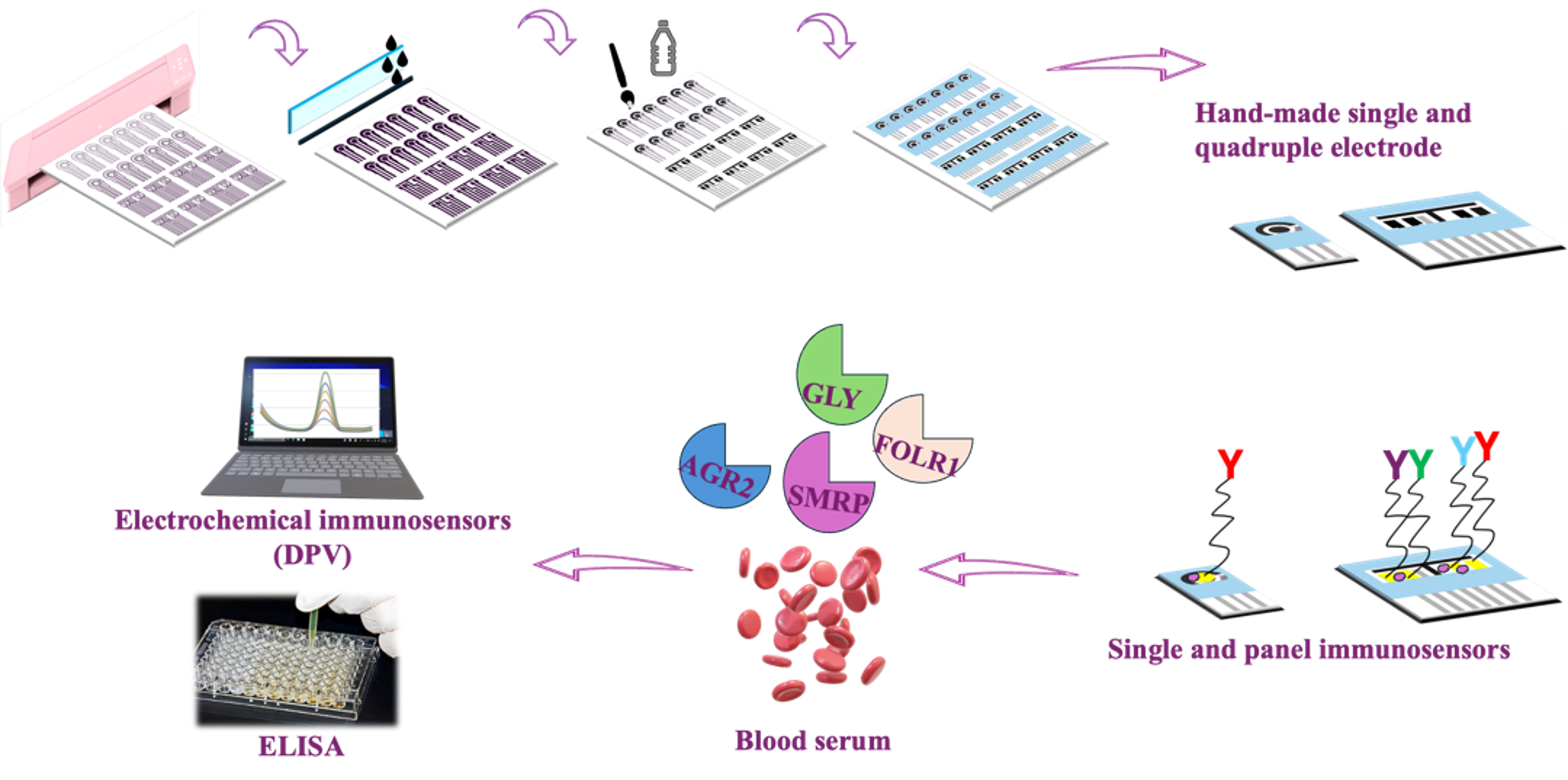
Graphical Abstract**
